# Archaic chaperone–usher pili self-secrete into superelastic zigzag springs

**DOI:** 10.1038/s41586-022-05095-0

**Published:** 2022-07-19

**Authors:** Natalia Pakharukova, Henri Malmi, Minna Tuittila, Tobias Dahlberg, Debnath Ghosal, Yi-Wei Chang, Si Lhyam Myint, Sari Paavilainen, Stefan David Knight, Urpo Lamminmäki, Bernt Eric Uhlin, Magnus Andersson, Grant Jensen, Anton V. Zavialov

**Affiliations:** 1 Joint Biotechnology Laboratory, MediCity, Faculty of Medicine, University of Turku, Turku, Finland; 2 Department of Physics, Umeå Centre for Microbial Research (UCMR), Umeå University, Umeå, Sweden; 3 Division of Biology and Biological Engineering, California Institute of Technology, Pasadena, CA USA; 4 Department of Molecular Biology, The Laboratory for Molecular Infection Medicine Sweden (MIMS), Umeå Centre for Microbial Research (UCMR), Umeå University, Umeå, Sweden; 5 Department of Cell and Molecular Biology, Biomedical Centre, Uppsala University, Uppsala, Sweden; 6 Department of Life Technologies, University of Turku, Turku, Finland; 7 Present Address: Division of Medicine, Dentistry and Health Sciences, University of Melbourne, Parkville, Victoria, Australia; 8 Present Address: Department of Biochemistry and Biophysics, Perelman School of Medicine, University of Pennsylvania, Philadelphia, PA, USA

**Keywords:** Bacterial pathogenesis, Bacterial secretion, Biofilms, Pathogens, Cryoelectron microscopy

## Abstract

Adhesive pili assembled through the chaperone–usher pathway are hair-like appendages that mediate host tissue colonization and biofilm formation of Gram-negative bacteria^1–3^. Archaic chaperone–usher pathway pili, the most diverse and widespread chaperone–usher pathway adhesins, are promising vaccine and drug targets owing to their prevalence in the most troublesome multidrug-resistant pathogens^1,4,5^. However, their architecture and assembly–secretion process remain unknown. Here, we present the cryo-electron microscopy structure of the prototypical archaic Csu pilus that mediates biofilm formation of *Acinetobacter baumannii*—a notorious multidrug-resistant nosocomial pathogen. In contrast to the thick helical tubes of the classical type 1 and P pili, archaic pili assemble into an ultrathin zigzag architecture secured by an elegant clinch mechanism. The molecular clinch provides the pilus with high mechanical stability as well as superelasticity, a property observed for the first time, to our knowledge, in biomolecules, while enabling a more economical and faster pilus production. Furthermore, we demonstrate that clinch formation at the cell surface drives pilus secretion through the outer membrane. These findings suggest that clinch-formation inhibitors might represent a new strategy to fight multidrug-resistant bacterial infections.

## Abstract (web summary)

The Csu pili of the multidrug-resistant nosocomial pathogen *Acinetobacter baumannii* assemble into an ultrathin zigzag architecture secured by a clinch mechanism that provides the pilus with high mechanical stability and superelasticity.

## Main

Adhesive pili, or fimbriae, are hair-like surface appendages that mediate bacterial infection and biofilm formation. In Gram-negative bacteria, most adhesive pili are assembled from protein subunits through the chaperone–usher pathway (CUP)^1^. CUPs are subdivided into three groups comprising six major phylogenetic clades: alternative (α-fimbriae), classical (β-, γ-, κ- and π-fimbriae) and archaic (σ-fimbriae)^1^. The best-studied classical CUPs include rigid pili with a tip adhesin subunit (such as P and type 1 pili) as well as thinner flexible pili (such as Saf, Psa and F4) that are known to act as polyadhesins^2,3^. Whereas classical and alternative CUPs are restricted to β- and γ-Proteobacteria, archaic CUPs are much more prevalent and present in a wide range of phyla^1^. Archaic CUPs are promising vaccine and drug targets owing to their wide distribution in the most troublesome pathogens, including panantibiotic-resistant *A. baumannii* and *Pseudomonas aeruginosa*^1,4,5^.

The formation of a dense biofilm is an essential trait of *A. baumannii* as a nosocomial pathogen, as it confers fitness for survival and persistence on surfaces^4,6^. The formation of this biofilm is mediated by Csu pili assembled through the archaic chaperone–usher CsuC–CsuD pathway^4^. The Csu pilus comprises the major subunit CsuA/B that forms the pilus rod, adaptor subunits CsuA and CsuB, and the two-domain tip adhesin CsuE that binds to various substrates using exposed hydrophobic finger-like loops^7^. The rod-forming CsuA/B subunit deviates from the standard Ig-fold and adopts an unusual architecture featuring two prominent hairpins A′–A″ and B–B′ protruding from the β-sheet^8^. This twin hairpin structure is unique to archaic CUPs^8^. Furthermore, in contrast to thick and rigid fibres from classical and alternative CUPs, Csu pili are surprisingly thin^7^, suggesting a substantially different pilus architecture. Classical CUP rigid pili form quaternary structures by packing into a thick, hollow helical tube^9–11^ that can elongate and unwind to resist strong rinsing flows^12^. The molecular architecture and biomechanical properties of archaic pili are unknown. We therefore sought to obtain a structure of the Csu pilus rod using cryo-electron microscopy (cryo-EM) and measure the Csu pilus force–extension response using optical tweezers.

## Csu pilus rod architecture

**Fig. 1:**
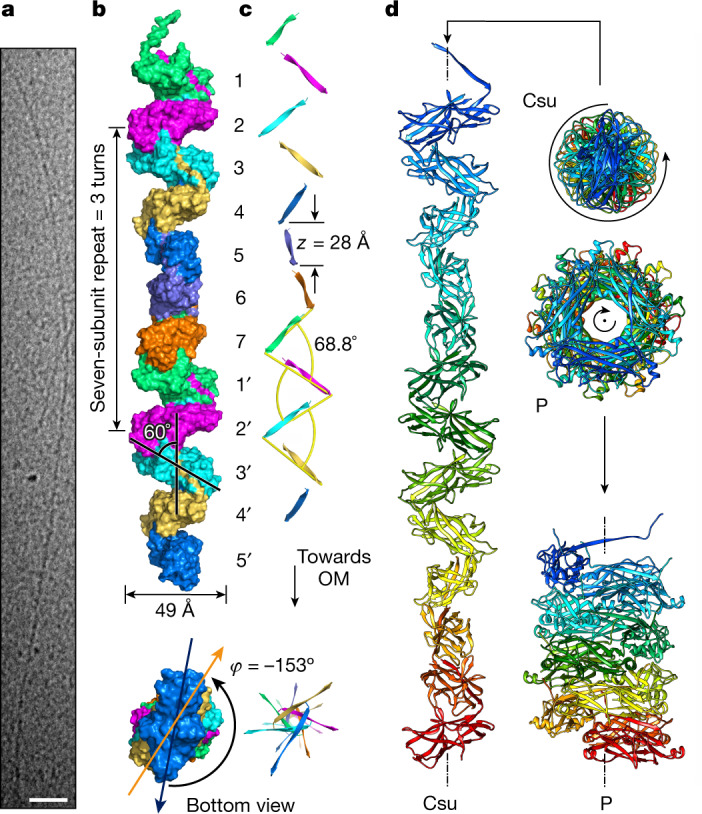
The Csu pilus rod is a thin zigzag-like filament. **a**, Cryo-EM image of the Csu pilus. Scale bar, 500 Å. **b**, Surface diagram of a 12-subunt fragment of the Csu pilus rod. Subunits are numbered in the direction of pilus growth, from the pilus tip to its assembly base at the outer membrane (OM). **c**, Cartoon diagram of the rod focusing on the donor strands. **d**, Cartoon diagrams of 13-subunit fragments of archaic Csu and classical P pilus rods. The zigzag filament is about three times as long as the helical tube rod. The handedness is indicated by a black curved arrow.

**Extended Data Fig. 1:**
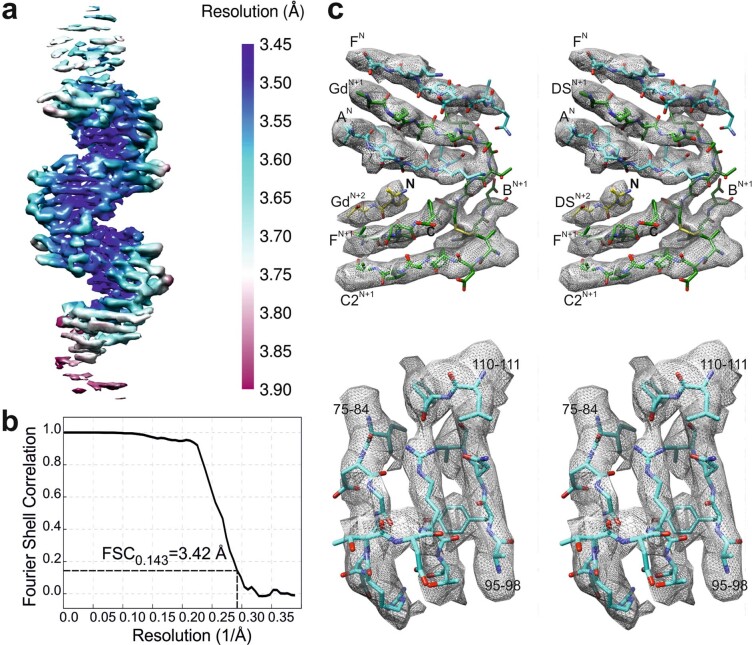
Resolution estimation and selected regions of the 3D electron microscopy map of the Csu pilus rod. **a**, Local-resolution estimation; the contour level of the map is 0.065. **b**, The Gold-standard Fourier shell correlation estimation at the 0.143 correlation threshold. **c**, Model fitting in density map (stereo view). Upper panel: Density for the interactive area between adjacent subunits CsuA/B^N^ and CsuA/B^N+1^, and Gd of subunit CsuA/B^N+2^ (Gd ^N+2^). Strands A^N^ and F^N^ in CsuA/B^N^, strands B^N+1^, C2^N+1^, and F^N+1^ in CsuA/B^N+1^ and donor strands Gd^N+1^ and Gd^N+2^ are labelled. Low panel: Density for a fragment of the subunit core, including residues 75–84, 95–98, and 110–111. The contour level of the map is 0.085.

Cryo-EM micrographs revealed thin and long, but remarkably stiff pili (Fig. 1a). The structure of the Csu pilus rod was determined to an overall resolution of 3.4 Å (Extended Data Fig. 1, Supplementary Table 1 and Supplementary Video 1). The Csu pilus is a thin (around 23 Å) left-handed filament with a helical rise (*z*) of 28.0 Å and rotation between subunits (*φ*) of −153° (Fig. 1b,c). Pilins are tilted around 60° relative to the helical axis and about 69° relative to each other, resulting in a zigzag appearance of pili when viewed in sideways projection. This architecture is substantially different from that of rigid classical pili^10^ (Fig. 1d). Notably, in Csu rods, the helical rise per subunit is three times longer than in P pili (Fig. 1d). Thus, the archaic assembly appears to be more economical and Csu pili are expected to grow in length three times faster than P pili at the same rate of pilin incorporation.

**Fig. 2:**
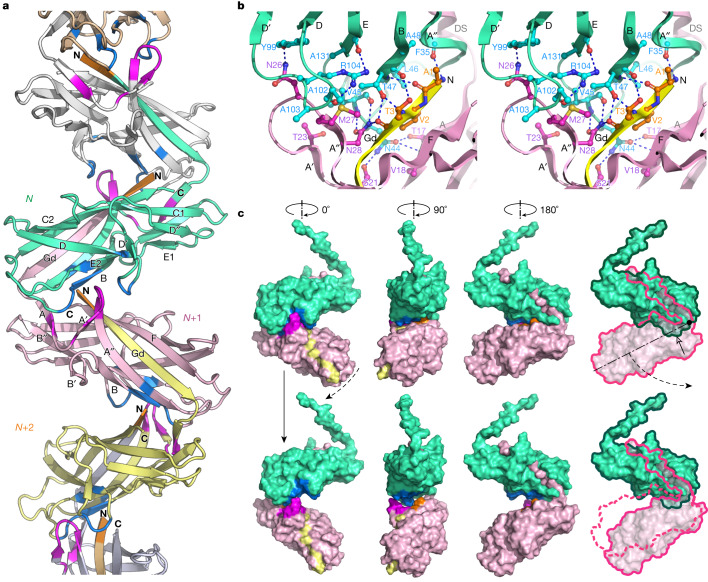
Csu pilus subunits are assembled by DSC and clinch mechanisms. **a**, Cartoon diagram of the rod. Clinch contact residues in the A strand and A′–A′′ hairpin, Gd donor strand N terminus and acceptor site are shown in magenta, orange and marine, respectively. The N and C termini as well as β-strands in the two central subunits are labelled. **b**, Stereo view of the clinch contact. Adjacent subunits CsuA/B^*N*^ and CsuA/B^*N*^^+1^, and Gd^*N*^^+2^ of subunit CsuA/B^*N*+2^ complementing CsuA/B^*N*+1^, are shown in green, pink and yellow, respectively. Interacting residues are shown as balls and sticks. The dashed lines represent hydrogen bonds. See also Supplementary Video 1. **c**, The structure of the clinch determines the pilus rigidity and trajectory of subunit movement during clinch formation or pilus stretching. The molecular surface of two adjacent subunits (green and pink) and Gd of the third subunit (yellow) is shown in three orientations obtained by viewing the structure after rotation around the pilus helical axis as indicated. Top, the fully closed structure. Bottom, a model of a partially opened conformation produced by rotating the lower subunit by about 17° around the linker. The residues involved in the clinch are coloured as described in **a**. The open arrowhead points at the surface buried between subunits. See also Supplementary Video 2.

**Extended Data Fig. 2:**
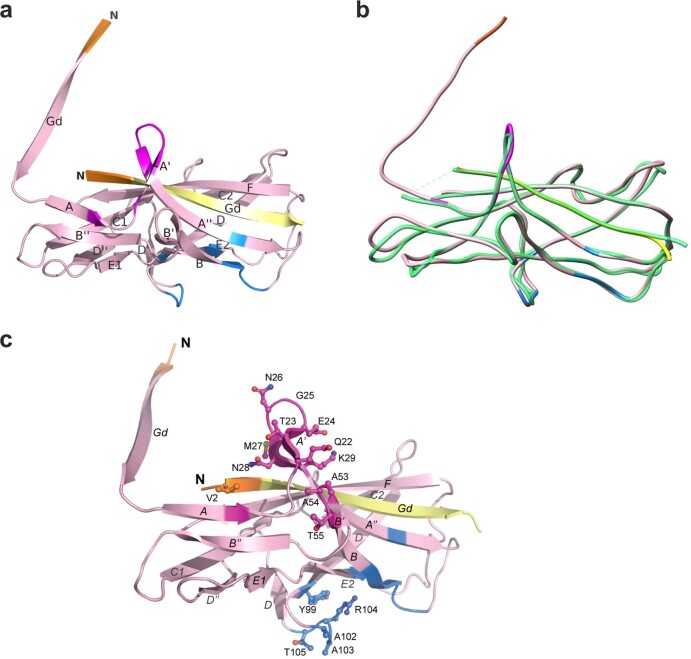
Structure of the CsuA/B subunit in the pilus rod. **a**, Cartoon representation of CsuA/B^N^ (pink) complemented by the Gd donor strand of the next subunit CsuA/B^N+1^ (yellow) in the rod. Clinch contact residues in the A strand and A′-A′′ hairpin, the N-terminus of donor strand Gd, and the acceptor site are painted magenta, orange, and marine, respectively. N-termini and β-strands are labelled. **b**, Superposition of the pilus CsuA/B^N^-Gd^N+1^ module with the crystal structure of self-complemented CsuA/B (CsuA/Bsc, PDB code 6FM5^8^, ribbon diagram). CsuA/B^N^-Gd^N+1^ is painted as in **a** and CsuA/Bsc is painted green. The dashed line indicates the unstructured linker in CsuA/Bsc that connects the Gd strand to the C-terminus. **c**, Cartoon representation of the CsuA/B subunit highlighting residues whose functional or structural roles or both were examined in this study (balls-and-sticks models). Colour coding is the same as in **a**, except that the entire twin-hairpin (A′-A′′ and B-B′ hairpins) is highlighted in magenta.

In contrast to their classical counterparts, the subunits in Csu rods are linked together not by one, but by two binding mechanisms. First, the incoming CsuA/B^*N*+1^ pilin subunit inserts its donor strand Gd^*N*+1^ into the hydrophobic groove of the preceding CsuA/B^*N*^ subunit (Fig. 2a). This is similar to donor strand complementation (DSC) in other CUPs^13–17^. The second binding mechanism, unique to archaic systems, involves an A′–A′′, B–B′ twin hairpin that protrudes like an arm from one side of CsuA/B (Fig. 2a and Extended Data Fig. 2). In the pilus, CsuA/B^*N*+1^ not only provides Gd^*N*+1^ to CsuA/B^*N*^, but also inserts its A′–A′′ hairpin into a pocket in CsuA/B^*N*^ between β-strands B and E2 and loop D–D′ (Fig. 2a,b). Moreover, the Gd^*N*+2^ of the third subunit CsuA/B^*N*+2^ that complements CsuA/B^*N*+1^ binds through its protruding N-terminal part to β-strands A′′ and B in CsuA/B^*N*^. Thereby, CsuA/B^*N*^ becomes firmly clinched between two extended surfaces of the CsuA/B^*N*+1^–Gd^*N*+2^ module (Fig. 2a–c): one from the A′–A′′ hairpin (Fig. 2c (magenta)) and the other from the N-terminal part of Gd^*N*+2^ (Fig. 2c (orange)). Finally, residues in β-strand A and loop A–A′ in CsuA/B^*N*+1^ form several contacts with residues in the A′′–B loop at the bottom of CsuA/B^*N*^, thereby bridging the two main binding sites to form a continuous binding surface of 600 Å^2^ with 41 interacting residues and 13 hydrogen bonds (Fig. 2b and Supplementary Video 1). Together, the clinch contact provides nearly one-third of the total interactive surface (32%) and hydrogen-bond network (31%) between pilins.

The linker between Gd and the globular domain of a pilin is flexible, but the A′–A′′ hairpin and Gd N terminus restrict the rotation of pilins to an up-and-down movement (Fig. 2c and Supplementary Video 2). Thus, the clinch contact confers pilus rigidity and determines the trajectory of subunit movement during clinch formation or pilus stretching (Fig. 2c).

## Csu pili are superelastic zigzag springs

**Fig. 3:**
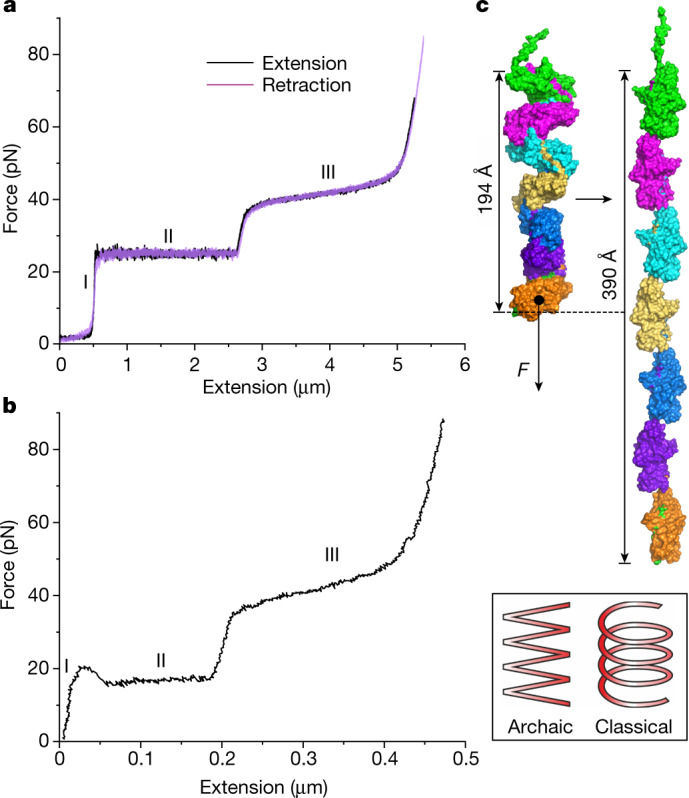
The archaic pilus rod is a molecular zigzag spring that exhibits superelastic properties. **a**,**b**, Examples of force–extension and retraction measurements of individual WT (**a**) and Val2Ala (**b**) Csu pili using optical tweezers. Note the different scales for the extension. The roman numerals (I–III) indicate the three regions defined in the force–extension (black) and retraction (purple) curves. Additional examples and control tests with non-piliated bacteria are shown in Extended Data Fig. 3. **c**, Csu pili can be extended to twice their length by clinch opening. Surface diagrams are shown of a seven-subunit helical repeat of the rod and a model of the maximally extended fibre. Inset: schematic of the conceptual difference in the molecular spring design between archaic (zigzag) and rigid classical (helical) pilus rods. *F*, force.

**Extended Data Fig. 3:**
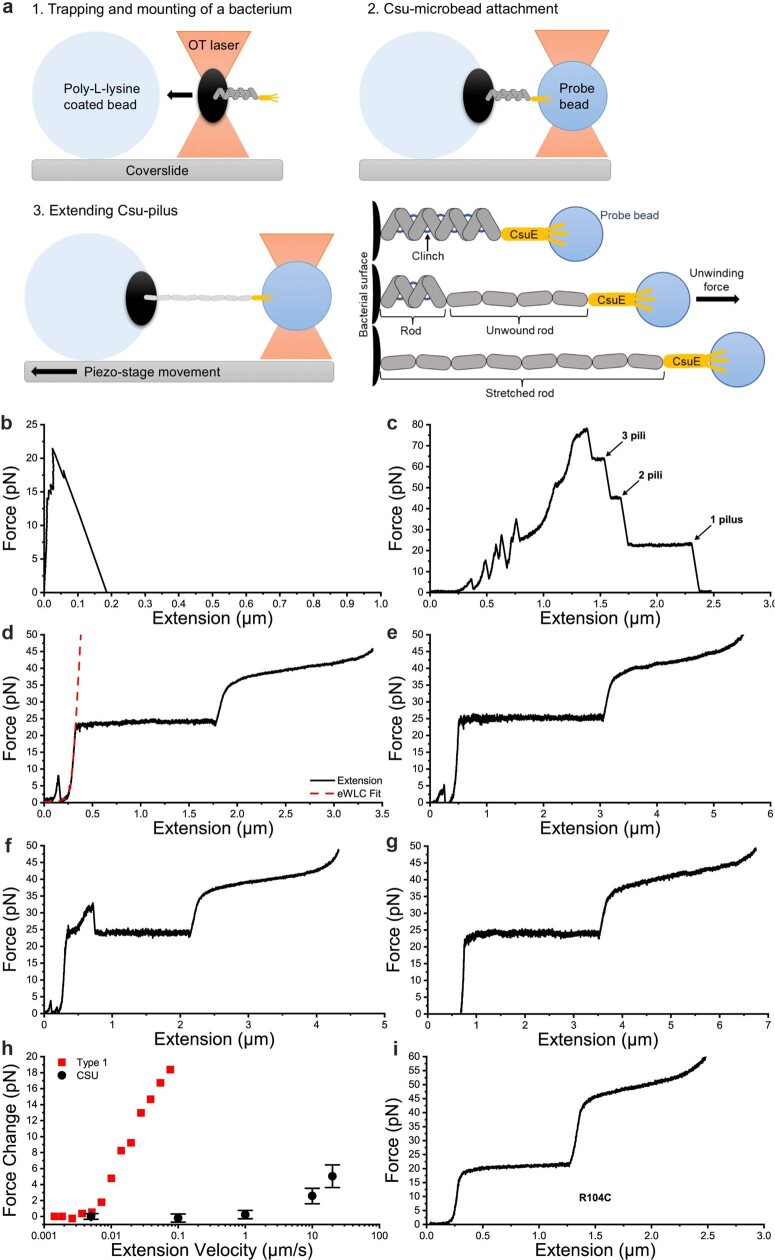
Force–extension measurement protocol and curves showing results from a non-piliated control, multi-pili interactions, and single pilus responses. **a**, Mounting and measuring procedures. 1) A 10 µm poly-L-lysine coated bead is immobilized on the coverslide, serving as a mount for a bacterium. A bacterium is trapped by the optical tweezers and attached to the coated bead. 2) A plastic probe bead is trapped, and the trap stiffness is calibrated using the active power spectrum method^45^. Subsequently, the probe bead is gently brought in proximity (about 1-2 µm) to the mounted bacterium and moved tangentially to the bacterium using a piezo-stage. Once a pilus attaches, the probe bead moves from the equilibrium position, which is observed in the live camera image and the real-time force response. 3) With a pilus attached, the piezo-stage is moved at 0.1 µm/s while keeping the trap stationary, resulting in a separation of the bacterium and probe bead complex. 4) A schematic illustrating Csu pilus extension under tensile force in region II. Top, the probe bead is attached, and the clinches are closed. Middle, half of the subunits in the pilus are unwound. Bottom, the whole pilus is unwound into a linearized filament. **b**, Example of a control measurement in which the probe bead was pushed into contact with a non-piliated bacterium (BL21-AI *E. coli* strain carrying an empty expression vector). Typically, the force only increased linearly before detachment, which occurred already at 0.02 µm extension, and with no indication of the probe bead extruding tethers from the membrane. **c**, Example of a force–extension curve resulting from multi-pili interactions. This may happen, if the probe bead is brought too close to a bacterium with multiple pili. Initially, the force response appeared chaotic, after which (at around 1.4 µm extension) three pili were stretched simultaneously into region II, giving rise to discrete unwinding force responses. Their combined force response was a multiple *n* x 23 pN. At 1.75 µm extension, only the longest pilus remained attached, and at 2.3 µm extension, the last pilus detached from the bead. **d**, Example data including a calculated wormlike chain (extensible wormlike chain model (eWLC); dotted red line) fit to region I of a WT pilus force curve. **e**-**g**, Three additional examples of force curves from individual WT pili. In **f**, the beginning of region II shows a force bump originating from a concurrent interaction with a shorter pilus that detached at 0.7 µm extension. **h**, Dynamic force spectroscopy data showing how the unwinding force increased in response to an increase in extension velocity (black dots, error bars represent one standard deviation, n = 5). The red squares are type 1 pilus dynamic data adapted from ref. ^23^. **i**, Example of a force–extension curve from a R104C mutant pilus.

The biomechanical properties of individual Csu pili were examined using force spectroscopy with optical tweezers (a schematic of the assay is shown in Extended Data Fig. 3a). As our previous biofilm quantification indicated that Csu pili efficiently bind to polystyrene using tip fingers^7^, we used polystyrene microbeads as probe beads in our measurements. Before performing these measurements, we rubbed the surface of cells lacking pili with a probe bead to confirm that no force response came from membrane tethers. None of the control cells displayed any tethers (Extended Data Fig. 3b). We could therefore assess the Csu force response under extension (black curve), which comprises the three regions previously reported for rigid classical P pili^18,19^ (Fig. 3a and Extended Data Fig. 3). Initially, the force increases linearly with extension, representing an elastic stretching of the rod (region I). Then, the force is constant with extension (region II). This region is interpreted as a sequential unwinding of the quaternary helical conformation of the rod. Thus, in Csu pili, it should correspond to a linearization of the zigzag filament. Finally, the force increases again linearly and shifts to a sigmoidal shape representing elastic stretching and presumably a conformational change in the pilins (region III)^19,20^. When reversing the movement and allowing the pilus to rewind, classical and archaic pili show a substantially different retraction response. Previous studies demonstrate that rigid classical CUP pili exhibit a dip in force associated with slack in the pilus needed to restore the helical structure^21^. By contrast, the contraction response of the Csu pilus (Fig. 3 (purple curve)) perfectly tracks that of the extension, reminiscent of shape memory metals that regain their original shape after deformation by external stress. Thus, the archaic Csu pilus acts like a superelastic molecular zigzag spring.

Analysis of the region I force responses by fitting an extensible wormlike chain model—which takes into account entropic bending fluctuations and enthalpic stretching—revealed that Csu pili have a persistence length of 0.89 ± 0.17 µm and a stretch modulus of 870 ± 120 pN (mean ± s.d.), suggesting a straight structure with high stiffness (Table 1; an example of extensible wormlike chain fit to region I is shown in Extended Data Fig. 3d). Using the persistence length and stretch modulus, we also calculated the pilus spring constant of region I and the flexural rigidity (bending stiffness; Table 1).

**Table 1:** Summary of pilus properties

Property^a^	WT	Val2Ala	Arg104Cys
Contour length (μm)	1.7 ± 0.6, *n* = 10	NE	1.2 ± 0.3, *n* = 10
Persistence length (μm)	0.89 ± 0.17, *n* = 16	NE	0.27 ± 0.07, *n* = 10
Stretch modulus (pN)	870 ± 120, *n* = 16	NE	460 ± 80, *n* = 10
Spring constant (pN nm^−1^)	0.51 ± 0.3, *n* = 16	NE	0.38 ± 0.17, *n* = 10
Flexural rigidity (N m^2^)	3.7 ± 0.7 × 10^−27^, *n* = 16	NE	1.1 ± 0.3 × 10^−27^, *n* = 10
Unwinding region II (μm)	1.7 ± 0.2, *n* = 36	0.24 ± 0.05, *n* = 18	1.2 ± 0.2, *n* = 10
S region III (μm)	2.1 ± 0.6, *n* = 10	0.36 ± 0.10, *n* = 8	1.3 ± 0.2, *n* = 10
Clinch opening force (pN)	23 ± 0.2, *n* = 41	15 ± 0.4, *n* = 22	19.8 ± 0.2, *n* = 10

NE, not estimated because of the short length of Val2Ala pili.

^a^The clinch opening force (region II) was measured from 41 individual WT pili. As some of these pili detached from the bead before complete extension to the end of region III, less data curves were available to measure contour length, persistence length and unwinding region length. As a consequence, the sample size (*n* values) for different force–extension parameters varies. Data are mean ± s.d.

Notably, the mean length of region II is identical to the estimated length of an average pilus (Table 1). Thus, the Csu zigzag filament reversibly unwinds to a linear conformation that is exactly twice its length. This almost integer elongation ratio (2.01) is also revealed by modelling and is the result of the peculiar geometry of the pilus: opening the clinch changes the tilt of the pilin from about 60° to around 0° or from cosine ∼0.5 to ∼1 (Fig. 3c). The Csu zigzag filament is less extendible than helical tubes such as P pili, which can unwind to around six times their original length^22^. However, Csu pili can be stretched much further at higher forces (region III). In P pili, this process is associated with unfolding of an α-helix in the rod subunit and subunit stretching^20^, indicating the possibility that CsuA/B may undergo large conformational changes at these forces.

The average force required to open the clinch contact is similar to unwinding forces for many tightly packed helical tube pili^22^ (Fig. 3). This notable coincidence suggests that the two different architectures both evolved to adapt to similar shear forces. However, Csu pili are more dynamic than their classical counterparts. Whereas the unwinding force for classical type 1 pili rapidly increases at unwinding velocities of greater than 0.006 μm s^−1^, resulting in a 20 pN increase in the force already at 0.1 µm s^−1^ (ref. ^23^), the force response of Csu pili remains relatively unchanged up to 20 μm s^−1^, increasing by only approximately 5 pN (Extended Data Fig. 3h). The rapid response of the Csu zigzag filament to the extension force is probably due to their linearized quaternary structure, in which subunits have only interactions with their nearest neighbours. By contrast, in helical tubes of P pili, each pilin interacts with ten other subunits, which greatly reduces the unwinding rate, restricting the ability to respond to sudden changes or fluctuations in a fluid flow rate^23^. Thus, archaic zigzag filaments can potentially mediate bacterial attachment in turbulent environments.

## Clinch formation drives pilus secretion

**Fig. 4:**
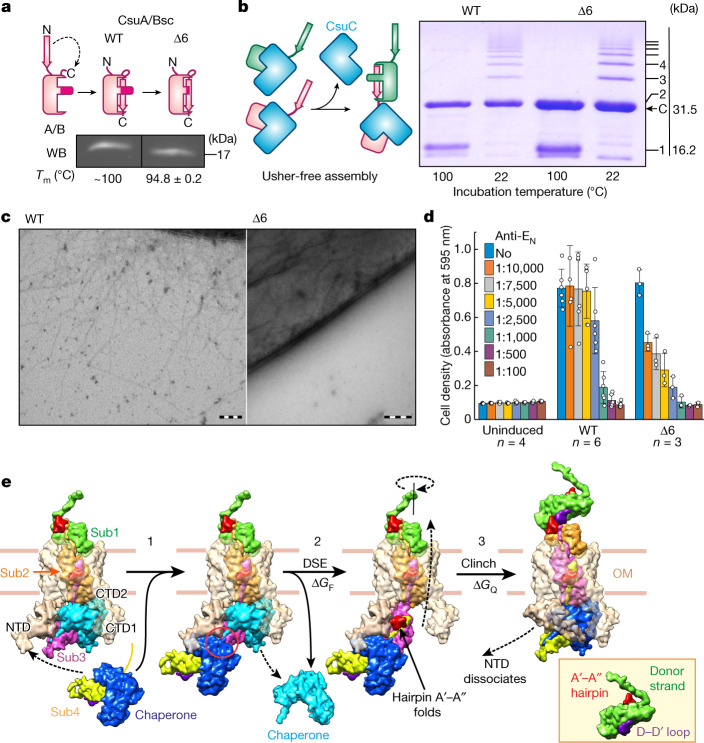
Clinch contact is required for efficient secretion of the pilus rod to the cell surface. **a**, Schematic of WT and Δ6 CsuA/Bsc constructs and western blot (WB) analysis of the periplasm extracted from *Escherichia coli* expressing these constructs. The melting temperature (*T*_m_) of the purified proteins is indicated below (Table 1 and Extended Data Fig. 6). **b**, Schematic of CsuC-assisted assembly of CsuA/B and SDS–PAGE analysis of CsuC–(CsuA/B)_*n*_ complexes purified from the periplasm of cells co-expressing CsuC with WT or Δ6 CsuA/B and pre-incubated at 22 °C or 100 °C. The positions of CsuC (C, 31.5 kDa) and (CsuA/B)_*n*_ with *n* = 1–8 are indicated (16.2*n* kDa). **c**, Negative-stained TEM micrographs of *E. coli* containing the WT or Δ6 *csu* gene cluster. Scale bars, 200 nm. **d**, Biofilm formation by WT or Δ6 *csu* *E. coli* at different concentrations of anti-tip (anti-CsuE NTD (anti-E_N_)) antibodies. Data are mean ± s.d. Data for individual wells are shown with open circles and the number of wells (*n*) is indicated. Analysis of other mutants is shown in Extended Data Figs. 4–7 and the data are summarized in Extended Data Table 1. **e**, The Csu pilus assembly–secretion cycle (Extended Data Fig. 8). The Δ*G*_F_ folding energy and Δ*G*_Q_ free energy of quaternary structure formation preserved by the chaperone and usher drive assembly and secretion, respectively (Supplementary Video 3). Western blot and gel source data are shown in Supplementary Fig. 1b,c.

**Extended Data Fig. 4:**
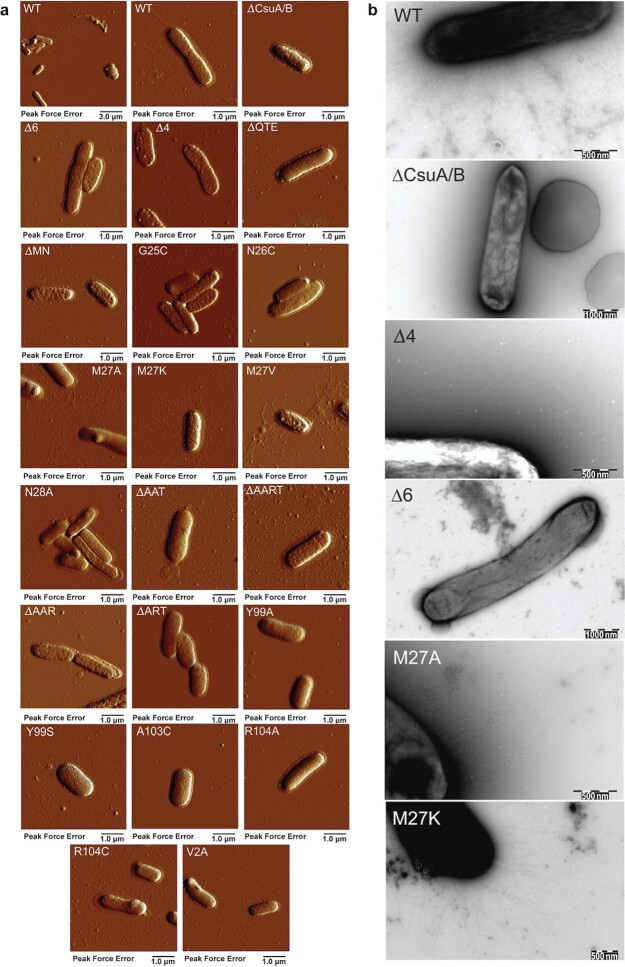
Analysis of morphology of Csu pili on the bacterial cell surface. **a**, Atomic force microscopy images of *E. coli* expressing the WT and mutant Csu pili. **b**, Negative stain transmission electron microscopy (TEM) micrographs of *E. coli* expressing the WT and mutant Csu pili. Cells were cultured in LB medium in the presence of 0.02% arabinose. For the TEM imaging, specimens were stained with uranyl acetate. Mutations are explained in Extended Data Table 1. For higher resolution images see source data in Supplementary Fig. 3.

**Extended Data Fig. 5:**
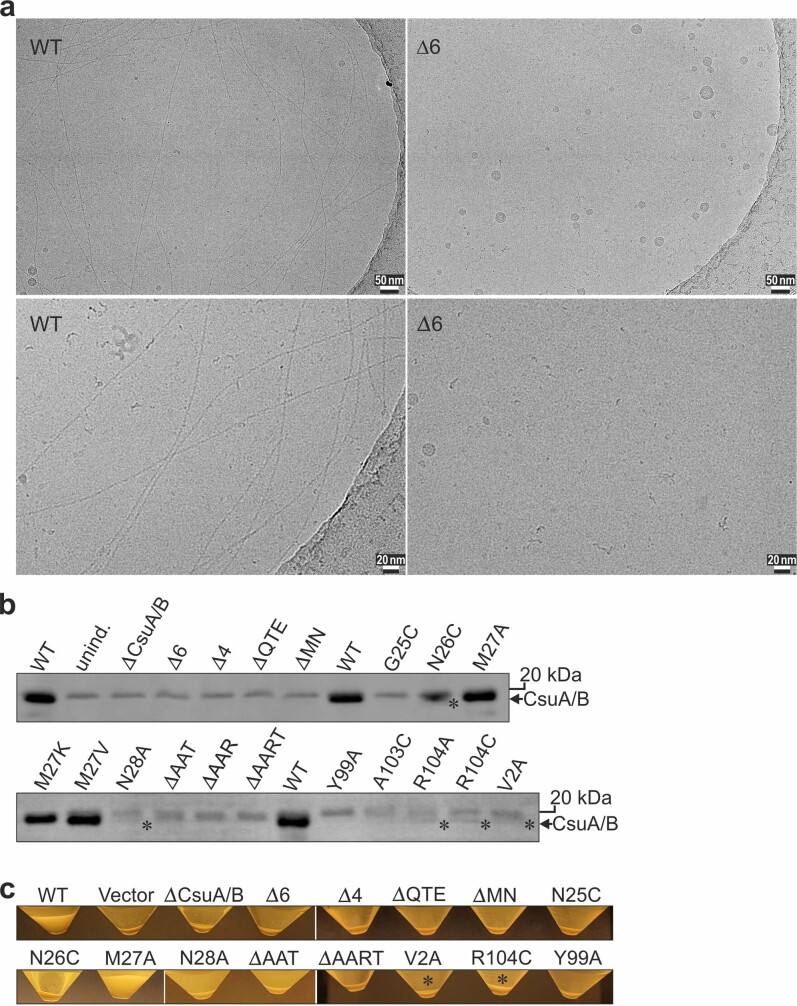
Effects of mutations on Csu pilus secretion and Csu-mediated resistance to precipitation. **a**, Cryo-EM analysis of surface-sheared pili obtained by the heat treatment of *E. coli* carrying the WT and Δ6 *csu* gene cluster. Micrographs were recorded with defocus of −3.5 μm on a Talos Arctica microscope at a magnification of 22,000 (upper images) and 45,000 (lower images). **b**, Western blotting of surface-sheared pili obtained by heat treatment of *E. coli* harbouring either WT or mutant *csu* gene cluster. CsuA/B was detected with rabbit polyclonal anti-CsuA/Bsc antibody and secondary IRDye 68RD5-conjugated anti-rabbit goat antibody. Note that the antibody also reacts with a protein moving slightly slower than CsuA/B. This band is not related to CsuA/B since it is present in extracts obtained from the ΔCsuA/B mutant and uninduced cells. While M27 mutants show CsuA/B levels similar to that of WT, no or very small amounts of secreted CsuA/B (<10% compared to WT, bands indicated by stars) are detected for other mutants. This correlates with cell imaging results (Extended Data Fig. 4) except for the mutant N26C, for which no pilus-like structures were observed despite the presence of a small quantity of CsuA/B in the extract. For WB source data, see Supplementary Fig. 1a. **c**, Influence of mutations on the Csu-mediated resistance of bacteria to sedimentation. Csu pili provide bacteria with a strong “parachute effect” that makes them highly resistant to sedimentation. Upon centrifugation, bacterial cells expressing WT pili form a soft pellet that is several times larger in volume than the dense pellet formed by control cells harbouring an empty expression vector (pBAD-ESNPA). The dense pellet is also observed for all our mutants except for point mutations of M27 that show the WT phenotype (exemplified here by M27A), and R104C and V2A that show a slightly softer pellet (marked by a star). The same number of bacterial cells of WT and mutant variants was used in experiments in **a**-**c**. Mutations are explained in Extended Data Table 1.

**Extended Data Fig. 6:**
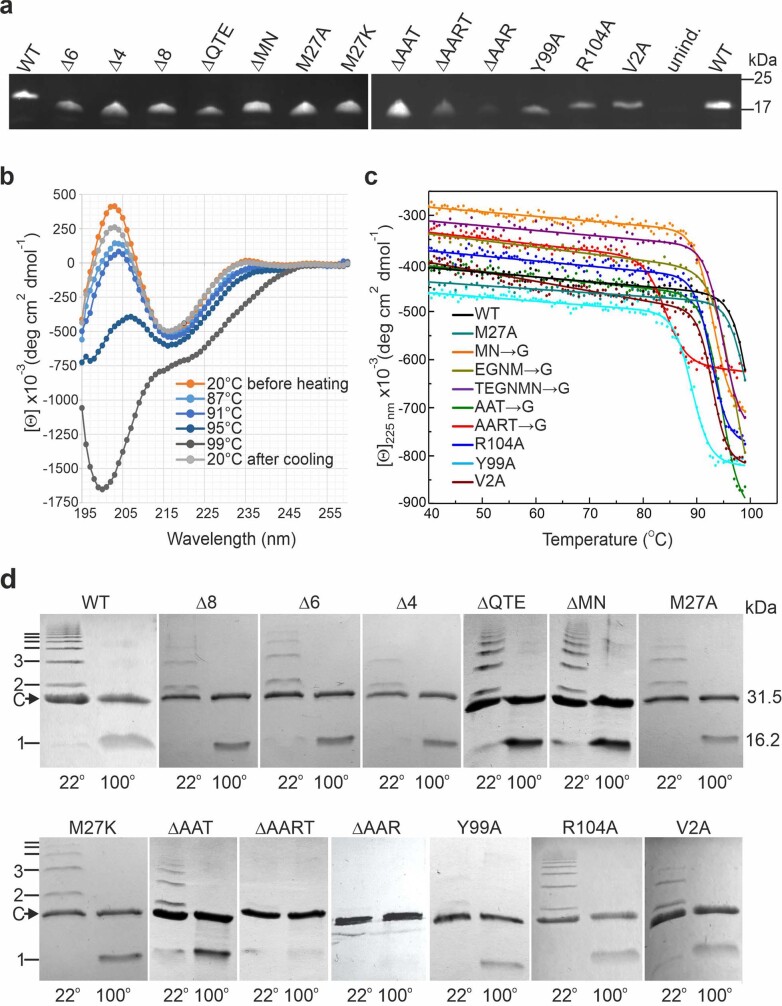
Effect of mutations on the stability of donor strand complemented CsuA/B and the ability of CsuA/B to form DSC-polymers. **a**, Western blotting of periplasmic extracts of *E. coli* expressing WT and mutant CsuA/Bsc. Periplasmic extracts were obtained from an equal number of cells. CsuA/Bsc was detected with rabbit polyclonal anti-CsuA/Bsc antibody and secondary IRDye 68RD-conjugated anti-rabbit goat antibody as described in Methods. **b**, CD spectra of WT CsuA/Bsc at different temperatures. **c**, Temperature dependence of molar ellipticity of WT and substituted variants of CsuA/Bsc at 225 nm. Melting temperatures generated from the data are listed in Extended Data Table 1. **d**, Effects of mutations on the ability of CsuA/B to form DSC-polymers. WT and mutant variants of CsuA/B were co-expressed with His_6_-tagged CsuC in *E. coli*, co-purified from periplasmic extracts by Ni^2+^-affinity chromatography, and analysed by SDS-PAGE. Complexes were incubated at 22 or 100 °C prior to the electrophoresis. Bars indicate positions of the melted CsuA/B monomer (1; 16.2 kDa) and six short CsuA/B polymers; the arrow shows the position of the CsuC chaperone (31.5 kDa). Mutations are explained in Extended Data Table 1. For WB and gel source data, see Supplementary Fig. 1b and d.

Considering the large interactive area of the clinch, we hypothesized that the clinch or its formation might have additional functions, and assessed its role in pilus stability, assembly and secretion by mutagenesis. First, we replaced a large portion of the A′–A′′ hairpin (residues TEGNMN; Extended Data Fig. 2c) with a single glycine (Δ6; Extended Data Table 1 and Supplementary Table 2). The Δ6 mutant of the self-complemented version of CsuA/B subunit (CsuA/Bsc) showed similar levels of expression and thermal stability to wild-type (WT) CsuA/Bsc (Fig. 4a, Extended Data Table 1 and Extended Data Figs. 4–6). Moreover, the deletion had no effect on the usher-free assembly of CsuA/B polymers in the periplasm (Fig. 4b). However, the Δ6 mutation completely abolished pilus expression on the cell surface, as observed by imaging of both cell-associated pili (Fig. 4c and Extended Data Fig. 4) and surface-sheared pili (Extended Data Fig. 5a), suggesting that the deletion disrupted pilus translocation through the usher channel. Similarly, shorter deletions within the A′–A′′ hairpin (MN to G, QTE to G, EGNM to G) did not affect subunit stability or assembly, but prevented pilus secretion (Extended Data Table 1 and Extended Data Figs. 4–6). A deletion in the B–B′ hairpin (AAT to G) also practically abolished secretion, although it forms no direct contacts with the neighbouring pilin. This result highlights its role in maintaining the binding-competent conformation of the A′–A′′ hairpin. Deletions in the D–D′ loop (AART to G, AAR to G, ART to GG) that forms a major part of the acceptor site for the A′–A′′ hairpin similarly prevented surface expression of Csu pili, although they also notably decreased subunit stability, resulting in less efficient usher-free assembly. Residue substitutions in the same region showed higher stability compared with the deletion mutants, yet no pili (Tyr99Ala/Ser) or only very small amounts of pilus-like material (Arg104Ala) were observed on the cell surface. Furthermore, substitutions in key residues of the A′–A′′ hairpin and acceptor site (Asn28, Asn26, Gly25 and Ala103), except for the partially buried Met27, abolished or severely inhibited secretion, permitting the assembly of only a few pilus-like structures on some bacteria. Consistent with EM and atomic force microscopy (AFM) imaging data, analysis of heat-detached (surface-sheared) WT and Met27-mutant pili using western blotting revealed a thick band of CsuA/B, whereas no or reduced amounts of CsuA/B were detected in surface-sheared pili of other mutants (Extended Data Fig. 5b). Csu pili provide bacteria with a strong ‘parachute effect’, a characteristic ability of piliated cells to resist sedimentation, which serves as an additional control for pili secretion (Extended Data Fig. 5c). As expected, all clinch mutations, except for Met27Ala, either substantially reduced (Arg104Cys and, to a greater degree, Val2Ala) or essentially abolished the parachute effect, further suggesting the role of the clinch contact in pilus secretion.

**Extended Data Fig. 7:**
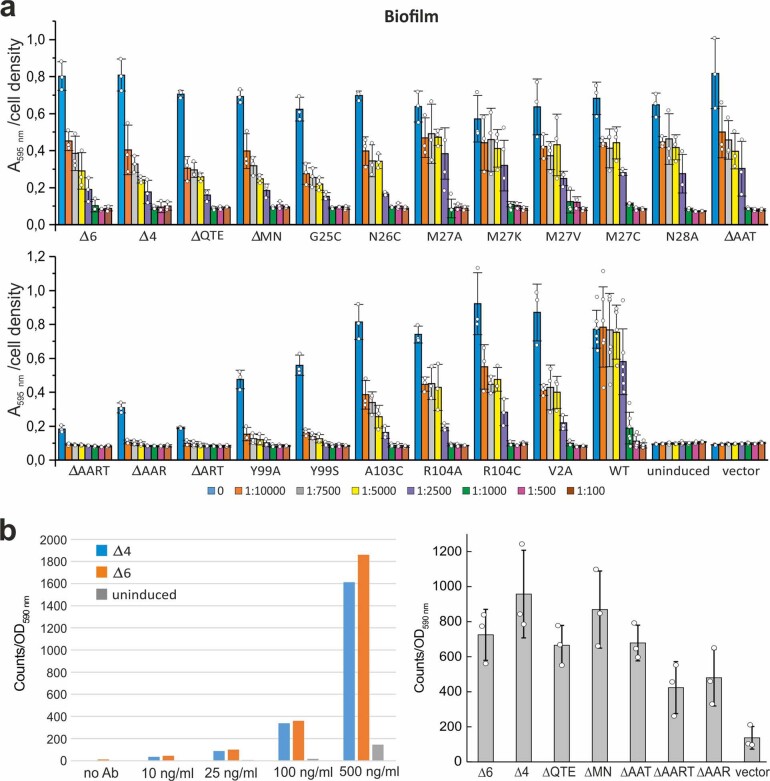
Effects of mutations on Csu-mediated biofilm formation and CsuE exposure on the cell surface. **a**, Quantification of biofilms formed in polystyrene microtiter plates by *E. coli* harbouring the WT or mutant *csu* gene cluster at different concentrations of the anti-tip antibody. Uninduced cells and induced cells harbouring an empty expression vector were used as a negative control. The data are represented as mean ± SD. Data for individual wells are shown with open circles (WT, n = 6; controls, n = 4; mutants, n = 3). **b**, CsuE is exposed on the cell surface despite mutations preventing subunit clinching in the pilus rod. Recombinant *E. coli* harbouring the *csu* gene cluster containing deletions within clinch contact sequences (see Extended Data Table 1) were grown for two hours in the presence of arabinose to induce gene transcription. The culture was divided in 4 ml aliquots and incubated for one hour with different concentrations of polyclonal antibody raised against the N-terminal domain of CsuE subunit (anti-tip antibody or αE_N_) labelled with Eu^3+^-chelate [N1-(4-isothiocyanatobenzyl)-diethylenetriamine-N1,N2,N3,N3-tetrakis(acetato)-europium(III)] (left panel) or with 250 ng/ml labelled antibody in triplicates (right panel) in the presence of 1% bovine serum albumin. The bacteria were washed four times by centrifugation at 5000×g and resuspending in phosphate buffered saline (PBS). The cells, resuspended in 1 ml PBS, were mixed 1:1 with the Europium Fluorescence Intensifier solution (Kaivogen, Finland) and time-resolved Eu^3+^fluorescence was measured in a 96-well plate using a 1420 VICTOR Multilabel Counter (PerkinElmer). Cell optical density was measured on a spectrophotometer at 595 nm. The graphs show counts per optical density of cells. Data are mean values ± SD (n = 3 bacterial cultures). Uninduced cells or *E. coli* harbouring an empty vector (pBAD-ENSPA) were used as negative controls.

Notably, the mutations did not prevent biofilm formation (Fig. 4d and Extended Data Fig. 7a), although they made biofilms more susceptible to the inhibition by anti-tip (anti-CsuE N-terminal domain (NTD)) antibodies. Consistent with this finding, we detected the CsuE tip subunit on the surface of the mutant bacteria using Eu^3+^-labelled anti-tip antibodies (Extended Data Fig. 7b). This result suggests that some form of short pili or tip fibrillum—composed of subunits CsuA, CsuB and CsuE—may still assemble and present the tip-finger adhesion site on the cell surface. Notably, the observation that biofilms mediated by these short structures are more susceptible to inhibition with anti-tip antibodies suggests that optimal attachment requires the involvement of pilus rods. This indicates a possible role of biomechanical properties or rod-mediated interactions between bacterial cells in this process, prompting research on this topic.

To investigate the role of the Gd N terminus in clinch formation, we substituted Val2—which mediates an important hydrophobic contact between the interacting pilins—with alanine (Fig. 2b). The mutation was well tolerated and did not affect assembly, but resulted in atypical, apparently short pili (Table 1 and Extended Data Fig. 4a). The force–extension response of Val2Ala pili was similar in shape to that of the WT pili, but much shorter in all three extension regions (Fig. 3b). The seven-times shorter unwinding region II suggests that Val2Ala pili are seven times shorter than WT pili (Table 1). Furthermore, the opening of the mutation-impaired clinch contacts required a much lower tensile force. Thus, this mutation provides an intermediate case suggesting that the length of secreted pili correlates positively with the strength of the clinch contact. The Arg104Cys mutant represents another interesting intermediate case. Although the mutation Arg104Cys substantially decreased the total number of surface pili, these pili were longer and mediated a stronger parachute effect compared with other pili affected by mutations (Extended Data Figs. 4a and 5b,c). Arg104Cys pili showed a force–extension curve similar to that of WT pili, but with a notably lower unwinding force and shorter region II (Table 1 and Extended Data Fig. 3i). Thus, as in the case of Val2Ala, this mutation negatively affects both the tightness of the clinch contact and the length of the pilus quaternary structure. Taken together, clinch formation is coupled to pilus rod secretion and is necessary for efficient expression of Csu pili on the cell surface.

**Extended Data Fig. 8:**
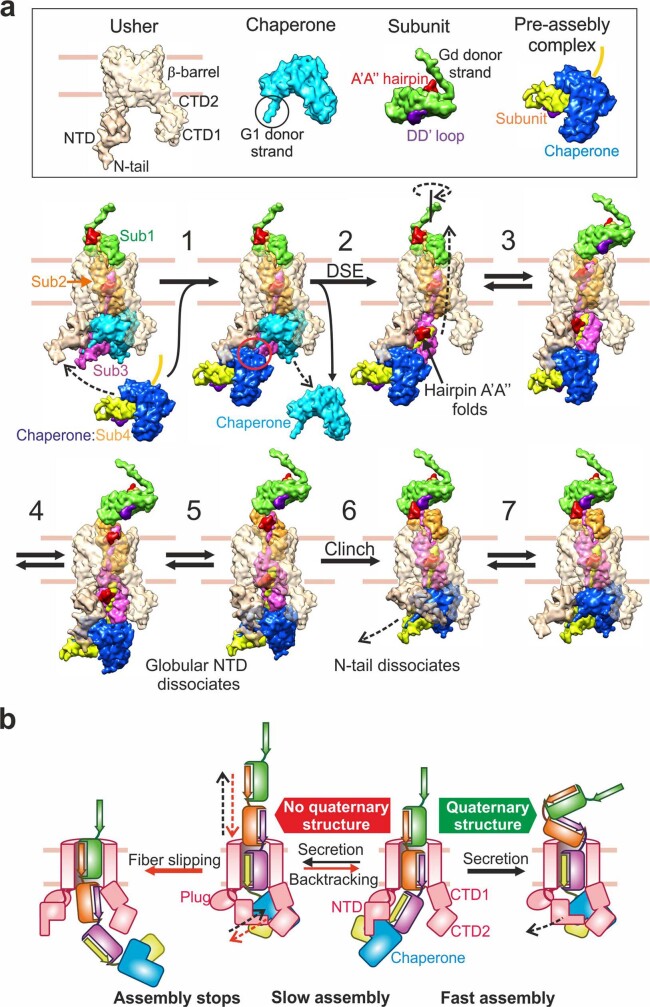
Csu pilus assembly-secretion mechanism. **a**, Assembly steps. (1) The CsuC-CsuA/B chaperone-subunit preassembly complex with CsuA/B partially stabilized by the G_1_ donor strand of CsuC^24^ binds to the usher NTD, while the base of a growing pilus represented here by a three-CsuA/B subunit fragment (Sub1-Sub2-Sub3) is in contact with the usher C-terminal domains CTD1 and CTD2. In classical CU systems, NTD mainly interacts with the chaperone using its N-terminal tail (N-tail) and surface residues of the globular domain, which contribute 60 and 40% of the binding surface, respectively^27^. (2) The Gd donor strand of the subunit in the preassembly complex (Sub4) replaces the G_1_ donor strand of the chaperone capping the base of the pilus in the zip-in-zip-out donor strand exchange (DSE) process^15,17^, linking Sub4 to the pilus. This also results in the complete folding of Sub3 and formation of the A′-A′′ and B-B′ hairpins^8^. The former pilus-capping chaperone is released. (3-4) In a reversible process, the pilus translocates up the usher channel. Due to the A′-A′′, B-B′ twin hairpin, movement within the usher is restricted to a relatively narrow path. To bring the Sub2 A′-A′′ hairpin closer to the acceptor pocket on Sub1, the secreted pilus outside the usher channel or (most probably) the usher has to rotate (see Supplementary video 3). (5) Still in a reversible process, Sub2, emerging from the usher secretion channel, leans to the edge of the usher bringing the Sub2 A′-A′′ hairpin closer to the Sub1 D-D′ loop. The globular domain of NTD dissociates from CsuC as the angle between these proteins becomes suboptimal for the interaction, while the flexible N-tail remains bound as is evident from the structure of FimD conformer 1^29^. (6) A clinch contact forms as Sub2 A′-A′′ hairpin and Sub3 Gd donor strand N-terminus bind to Sub1 from two sides, while Sub1 D-D’ loop covers the Sub2 A′-A′′ hairpin from the front, locking it with a second layer of interactions. The formation of the clinch is likely facilitated by narrowing down the number of pathways towards its formation: When Sub1 and Sub2 approach one another, the A′-A′′ hairpin and Gd N-terminus prevent sideways rotation of Sub1 as shown in Fig. 2c. Clinch formation links Sub2 to the rigid pilus stalk, making the subunit unable to re-enter the usher channel. This mechanism prevents pilus backtracking, driving unidirectional secretion. (7) Upon the completion of the secretion cycle, the N-tail of the usher NTD reaches CTD2 and loses its affinity to the chaperone as suggested by Du et al^29^. NTD is released to accept a new preassembly complex. **b**, Lack of a proper pilus rod quaternary structure may cause secretion backtracking and slipping of the fibre from the assembly platform. The pilus rod quaternary structure can potentially act as a ratchet-like mechanism preventing backtracking of the secretion step as well as hazardous accidents, in which the base slips away from the usher after its release from NTD, permanently jamming the assembly. See also Supplementary Video 3.

To understand how clinch formation may facilitate secretion, we modelled the assembly–secretion process based on the available structures from the Csu system and classical CUPs (Fig. 4e, Extended Data Fig. 8 and Supplementary Video 3). The incoming CsuC–CsuA/B preassembly complex^24^ is recruited to the usher NTD, while the chaperone-capped base of the growing pilus (represented by a Sub1–Sub2–Sub3 fragment) is positioned at the usher C-terminal domains CTD1 and CTD2^25–28^ (steps 1–2 in Fig. 4e and Extended Data Fig. 8). The Gd of chaperone-bound Sub4 replaces the G_1_ strand of the base chaperone through donor strand exchange (DSE), linking Sub4 to Sub3^15,16^. DSE results in the complete folding of Sub3 and formation of the A′–A′′ and B–B′ hairpins^8^. However, Sub3 cannot form a clinch contact with its neighbouring subunits, as Sub2 has entered the narrow usher channel and the chaperone-bound Sub4 is only partially folded and lacks its own twin hairpin^24^. The clinch contact can be formed only between subunits Sub1 and Sub2 on the cell surface (Fig. 4e (step 3)). Thus, the formation of the twin hairpin not before but after DSE has three purposes. First, it provides an additional folding potential to drive assembly^8^. Second, it prevents premature subunit clinching, thereby keeping the fibre in the elongated conformation required for secretion. Finally, it enables the formation of the quaternary structure immediately after subunit translocation.

**Extended Data Fig. 9:**
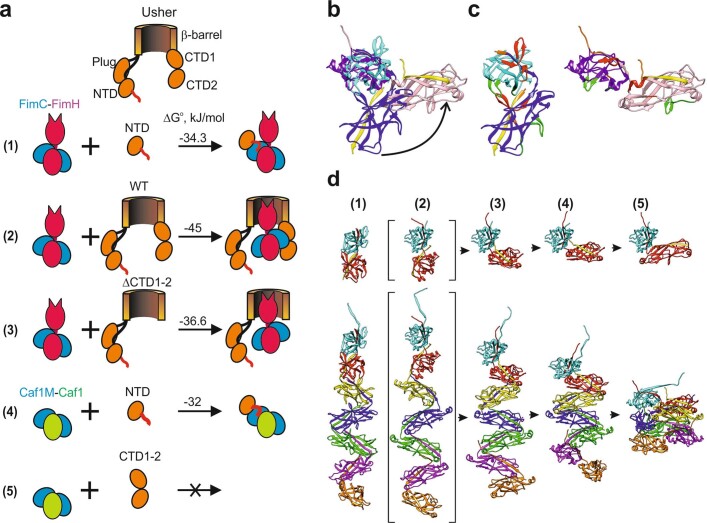
Thermodynamics of the pilus base handover and predicted evolutionary pathway from a zigzag to a helical tube architecture. **a**, Affinities of pre-assembly complexes to usher NTD and CTD1-2 in classical CUP systems. In classical CUPs, NTD mainly interacts with the chaperone using its N-terminal tail (N-tail, wavy line), resulting in a rather strong binding affinity. Binding of the FimC-FimH chaperone-adhesin subunit complex to NTD of the FimD usher from the Type 1 pili system (1) is characterized by *K*_*d*_ = 0.96 μM (*ΔG*^*o*^ = −34.3 kJ/mol)^46^ and binding of the Caf1M-Caf1 chaperone-subunit complex to NTD of the Caf1A usher from the F1 antigen system (4) by *K*_*d*_ = 2.4 μM (*ΔG*^*o*^ = −32 kJ/mol)^27^. However, no binding was detected between the Caf1M-Caf1 chaperone-subunit or Caf1M-Caf1-Caf1 complexes with either single CTD2^47^ or CTD1-2 constructs (5) (S. Roy and A. Zavialov, unpublished data). Although Werneburg et al. suggested the FimC-FimH complex has higher affinity for CTDs than NTD in the Type 1 pili system^48^, their excellent binding data seem to argue against this conclusion. The observed high affinity of the FimC-FimH complex to the usher (*K*_*d*_ = 12.5 nM, *ΔG*^*o*^ = −45 kJ/mol) (2) is probably determined by the interactions of the lectin domain of FimH with the usher channel (3) rather than binding to CTDs since the deletion of CTDs in their study reduced the affinity only moderately (30 times, *ΔG*^*o*^ = −8.4 kJ/mol). Besides, the structural analysis of the Type 1 system^26,28^ does not seem to reveal any particularly important hydrophobic interactions between the base and CTDs that would corroborate the existence of tight binding between them, whereas the N-tail and preassembly complex form a considerable ∼600 Å^2^ hydrophobic interface^27^. At the same time, it should be noted that obtaining accurate data in such a complex system involving a membrane protein is a challenging task. Hence, these calculations may change substantially when more accurate data become available. Furthermore, the role of this factor in driving pilus secretion can be different in different systems and relative contributions of different factors in different systems are currently not known. **b**, Fragments of Csu and Pap pilus rods consisting of two adjacent subunits (N and N+1) and the donor strand from the third subunit (Gd^N+2^) were superimposed over Cα atoms of the first subunits. CsuA/B^N^ and CsuA/B^N+1^ are shown in cyan and blue, PapA^N^ and PapA^N+1^ in violet and pink, and Gd ^N+2^ in yellow. A change from the Csu zigzag architecture to a tube-like helix would require the second subunit to rotate around 90° as indicated by an arrow. **c**, Same fragments as in **b** shown separately. The A′-A′′ hairpin, N-terminal part of the Gd strand, D-D′ loop, and other regions involved in the clinch contact between subunits in the Csu rod are shown in red, orange, green, and dark green, respectively. Corresponding regions in the PapA subunits have the same colours. **d**, Predicted evolutionary pathway from a zigzag to a helical tube architecture. The images show adjacent subunits as in (b) to illustrate changes in the inter-subunit angle (upper images) and seven-subunit pilus rod fragments to illustrate changes in the rod architecture (lower images). (1) The Csu pilus, a member of the archaic CUP fimbriae. The A′-A′′ hairpin locks the angle between subunits. (2) A hypothetical model obtained by superimposing PapA subunits over CsuA/B subunits in the Csu rod. This hypothetical model suggests that loss (or initial lack) of an A′-A′′ hairpin may have allowed subunits to rotate relative to each other to form a more compact helical packing. A longer N-terminal tail may have evolved to stabilize the inter-subunit contact in the absence of an A′-A′′ hairpin. (3) A hypothetical early intermediate. A′-A′′ hairpin (as in CsuA/B) changing into an α-helix (as in PapA) may have enabled a shift in the angle between subunits, while the N-terminal tail of Gd donor strand may have helped maintain stability. (4) A hypothetical late intermediate. Further change in the angle may have resulted in various open spiral architectures still lacking connections between the subunit layers. (5) Pap pilus, a member of the classical CUP fimbriae. The final step in the evolution would be the formation of interactions between subunit layers. At this stage, most structures stabilizing the open spiral architectures would have become unnecessary and disappeared.

The secretion step involves handover of the base from NTD to CTDs. The handover does not seem to be driven by the binding of the base to CTDs, as neither important hydrophobic interactions nor affinity between the base and CTDs have been observed (Extended Data Fig. 9a), questioning the origin of forces and energy driving secretion in CUPs. The FimD usher NTD was recently shown to escort the base until it reaches CTDs and forms interactions with CTD2 that could potentially facilitate the release of the base from NTD^29^. Our findings demonstrate that secretion of the pilus rod is greatly facilitated by quaternary structure formation, representing an alternative driving force. As the formation of a single clinch reduces the fibre length by exactly the length of one pilin, clinch formation may actively pull subunits to the cell surface without introducing shifts in their positions at each cycle (Extended Data Fig. 8a). Moreover, clinch formation may prevent backtracking of the secretion step that could potentially lead to the base slipping away from the usher after its release from the NTD, permanently jamming the assembly (Extended Data Fig. 8b). Future structural studies on the assembly–secretion mechanism of the CsuD usher will help to validate these hypotheses.

## Discussion

The clinch–DSC-based zigzag filament is probably the earliest and most widely used architecture of pili assembled through the CUP. This economical design gives the pilus a surprisingly high mechanical stability, rapid dynamic properties and superelasticity. The pilus secretion process involves an elegant mechanism that allows clinch formation only at the cell surface (Fig. 4e). Thus, similar to the chaperone that preserves folding energy of the subunit to drive pilus assembly, the usher inhibits the formation of the quaternary structure, preserving energy of intersubunit contacts to drive pilus secretion through the membrane. Notably, polymers of classical CUP subunits can easily adopt the zigzag filament architecture of archaic pili (Extended Data Fig. 9b–d), suggesting that both types of pili might follow a similar conserved secretion pathway before they reach the stage of forming the final quaternary structure. It therefore cannot be excluded that secretion of rigid helical pili might be partially driven by intermediate rather than the final intersubunit contacts, inviting further studies on this topic. The secretion process of the more flexible polyadhesive pili from the classical CUP as well as tip fibrillum structures also remains unclear. It might involve weaker quaternary interactions lacking high-resolution characterization. The elucidation of the structure and assembly–secretion process of ubiquitous archaic pili should pave the way for the development of clinch-formation inhibitors against persistent bacterial infections.

## Methods

### Bacterial strains and plasmids

*E. coli* strain DH5α was used for plasmid propagation. Protein expression was performed in *E. coli* BL21-AI (F^−^*omp*T *hsd*S_B_(r_B_^−^ m_B_^−^) *gal* *dcm* *ara*B::T7RNAP-*tet*A; Invitrogen). Expression plasmids were constructed based on pBAD-Csu aka pBAD-Csu(A/B)ABCDE^7^, pET101-6HCsuA/Bdsc^8^ and pET101-CsuC6H-CsuA/B^24^. Deletions and substitutions were generated using inverse PCR. Deleted codons were removed precisely using pairs of primers that corresponded to the sense strand downstream or the complementary strand upstream of the deleted sequence. To generate insertions and substitutions, oligonucleotides were designed to contain additional nucleotides or nucleotide changes, respectively. Amplified fragments were treated with DpnI, purified and blunt-end self-ligated before transformation of *E. coli* DH5α. To introduce mutations in pBAD-Csu, the NotI–SpeI fragment of the plasmid was first subcloned in the pGEM5, pSP72 or pBluescript vector to generate mutations by PCR, and then the original sequence in pBAD-Csu was replaced by the mutated fragment using the NotI and SpeI enzymes. Mutations were confirmed by sequencing of the intermediate and final constructs. A list of the oligonucleotides and generated plasmids is provided in Supplementary Table 2.

### Protein expression and purification

WT and mutant CsuA/B were co-expressed with the CsuC chaperone, carrying a C-terminal His_6_ tag, in the periplasm of *E. coli* containing the pET101-CsuC6H-CsuA/B-## plasmid series (Supplementary Table 2). WT and mutant CsuA/Bsc were expressed in the periplasm of *E. coli* containing the pET101-6HCsuA/Bdsc-## plasmid series (Supplementary Table 2). Periplasmic fractions were obtained by osmotic shock as described previously^30^. In brief, the pelleted cells were resuspended in 20% (w/v) sucrose in 20 mM Tris–HCl pH 8.0, 5 mM EDTA. After incubation for 10 min on ice, the cells were collected by centrifugation (7,000*g* for 15 min) and carefully resuspended in ice-cold 5 mM MgSO_4_. The cells were again pelleted at 7,000*g* for 15 min and the supernatant (that is, the periplasmic fraction) was collected. WT and mutant CsuC_His6_–(CsuA/B)_*n*_ complexes were purified from the periplasm by Ni-chelate chromatography essentially as described previously^24^. WT and mutant CsuA/Bsc_His6_ were purified by Ni-chelate chromatography as described earlier^8^, dialysed against 20 mM bis-TRIS propane, pH 9.0, and purified further by anion-exchange chromatography on the Mono Q 5/50GL column (GE Healthcare). For circular dichroism measurements, the buffer was exchanged to 12.5 mM potassium phosphate, pH 7.0 using a PD-10 desalting column (GE Healthcare). Protein concentrations were measured using the NanoDrop 2000 Spectrophotometer (Thermo Fisher Scientific).

To express WT and mutant variants of Csu fimbriae, *E. coli* BL-21-AI cells were transformed with ampicillin-resistant pBAD-Csu (pBAD-(CsuA/B)ABCDE) and its derivatives (Supplementary Table 2). Selected clones were cultivated in Luria–Bertani (LB) medium supplemented with 100 μg ml^−1^ ampicillin overnight at 37 °C and refreshed by 1:400 dilution of LB medium containing 80–100 μg ml^−1^ ampicillin. The cells were grown at 37 °C to an optical density at 600 nm of 0.8–1.0, then induced with 0.2% l(+)-arabinose for protein expression and grown for a further 2.5 h. The cells were collected by two rounds of centrifugation at 5,000*g* for 30 min and 7,000*g* for 10 min. The bacterial pellet was resuspended in 0.5 mM Tris-HCl, pH 7.4, 75 mM NaCl and incubated at 65 °C for 1 h. After incubation, the bacteria were pelleted by two rounds of centrifugation at 9,500*g* for 10 min. The supernatant containing detached Csu fimbriae was carefully collected and stored at 4 °C before analysis. Before cryo-EM analysis, the quality of the preparation was assessed by negative-stain transmission EM.

### Negative-stain EM analysis of purified pili

Purified Csu pili were applied on Formvar-coated glow-discharged gold grids (Agar Scientific) and incubated for 1 min. After blotting the excessive sample, the grid was washed with two drops of water, blotted again and then stained with 2% uranyl acetate. Images were acquired on the JEM-1400 Plus transmission electron microscope (JEOL) operated at 80 kV.

### Cryo-EM

Supernatant containing detached Csu fimbriae was concentrated to approximately 10 g l^−1^ using a Vivaspin device (Sartorius Stedim) with a molecular mass cut‐off of 100 kDa. Then, 4 µl of sample was applied to glow-discharged Quantifoil R2/2 300 mesh copper grids coated with ultrathin carbon (Electron Microscopy Sciences). The grids were blotted and plunge-frozen into liquid ethane using the Vitrobot Mark IV (Thermo Fisher Scientific) at 4 °C and 100% humidity. The data were collected on a 300 kV Titan Krios electron microscope (Thermo Fisher Scientific) equipped with a Gatan K3 direct electron detector operated in super-resolution mode with a pixel size of 0.433 Å and a defocus range of −1.0 to −3.0 μm. A total dose of 60 electrons per Å was applied and equally divided among 40 frames to allow for dose weighting. SerialEM (v.3.6) was used for automated cryo-EM data collection. Details on cryo-EM data collection are summarized in Supplementary Table 1. A representative cryo-EM micrograph of Csu pili is shown in Supplementary Fig. 2.

### Cryo-EM image processing and reconstruction

Dose-fractionated video frames were processed for beam-induced motion correction using MotionCor2 (v.1.2.3)^31^. CTF was estimated using CTFFIND (v.4.1.13). Image processing and helical reconstruction were performed in RELION (v.3.0.8)^32^. Filaments manually picked from 602 selected micrographs using the e2helixboxer program within EMAN 2^33^ were subjected to 2D classification to generate auto-picking templates. After autopicking of helical filaments, a total of 480,064 segments were extracted with a box size of 400 pixels. After the 2D and 3D classification steps, 255,833 segments were used for 3D refinement. The segments were rescaled to a pixel size of 1.35 Å. A starting model for reconstruction was generated de novo from the 2D particles using the stochastic gradient descent algorithm in RELION (v.3.0.8). Helical symmetry parameters were estimated using conventional Fourier–Bessel analysis and the segclassreconstruct and seggridexplore modules in SPRING (v.0.86)^34^. Initial estimates of helical parameters (−157° helical twist, 26.3 Å helical rise) were tested using a search range of −150° to −165° for the twist and 26 Å to 30 Å for the rise. The helical symmetry (−153° helical twist, 28 Å helical rise) was applied and refined during high-resolution 3D refinement producing a map with a resolution of 6.18 Å. Applying a soft mask with a raised cosine edge of 14 px and *B*-factor sharpening yielded a map with a global resolution of 4.8 Å as assessed using the gold standard Fourier shell correlation procedure between independently refined half reconstructions (FSC 0.143)^35^. The resolution was further improved to 3.42 Å after two iterations of Bayesian polishing followed by 3D refinement and post-processing. The final map showed clear β-strand separation and density for bulky side chains consistent with the reported resolution. The pixel size of the cryo-EM maps from RELION was slightly off and was adjusted to 1.2949 Å by calculating the range of cross-correlation coefficient values of the map with different voxel sizes to the refined model using the Fit in Map tool in the UCSF Chimera package (v.1.15)^36^.

### Model building and refinement

The initial model of the Csu pilus was built manually by fitting the crystal structure of CsuA/Bsc (Protein Data Bank (PDB): 6FM5)^8^ into the experimental electron density using UCSF Chimera. The angle between two subunits was adjusted using the Chimera Fit in Map tool in several iterations of first docking three subunit dimers into adjacent regions in the map with one subunit overlap and averaging the orientations of the overlapping subunits, then overlapping the three dimers fully and averaging the subunit orientations of all three dimers. The short linker connecting the donor strand with strand A was modelled using Coot (v.0.9.4)^37^. The structure was refined by combining manual adjustments in Coot and real space refinement in PHENIX (v.1.8.2)^38^. The initial four-subunit model was reduced to a model with three donor strand complemented subunits (four chains) that occupy the highest-resolution positions in the map. The model was validated using MolProbity (v.4.5.1)^39^. Refinement statistics are given in Extended Data Table 1.

### AFM

Bacteria were grown on the LB agar plate supplemented with ampicillin and induced with 0.02% arabinose to produce pili. The bacterial cells with pili were imaged by AFM as described earlier^40^ with some modifications. In brief, bacterial cells were suspended in 100 µl of Milli-Q water and 10 µl the suspension was placed onto a freshly cleaved mica surface (Goodfellow Cambridge). The samples were incubated for 5 min at room temperature and blotted dry before they were placed into a desiccator for a minimum of 2 h to dry. Images were collected using the Nanoscope V Multimode 8 AFM instrument (Bruker) using the Bruker ScanAsyst mode with the Bruker ScanAsyst-air probe oscillated at a resonance frequency of 50–90 kHz, selected using the Nanoscope (v.1.8) software. Images were collected in air at a scan rate of 0.8–1.5 Hz depending on the size of the scan and the number of samples (256 or 512 samples per image). The final images were plane-fitted in both axes and presented in amplitude (error) mode.

### Biofilm assay and biofilm inhibition

*E. coli* strain BL21 containing pBAD-Csu or its derivatives was cultured overnight in LB medium in the presence of 100 mg l^−1^ ampicillin. A total of 5 ml of the fresh medium in a 50 ml polypropylene tube was inoculated with 100 μl of the overnight culture and then grown at 37 °C with vigorous shaking for 2 h. Dilutions (100, 500, 1,000, 2,500, 5,000, 7,500 and 10,000 times) of the anti-CsuE N terminus polyclonal antibody (custom produced by Innovagen AB using purified CsuE N terminus)^7^ in 50 μl LB were divided into microtitre plate wells. Bacterial cultures were induced with 0.2% arabinose, and 150 μl replicates were mixed with the antibody dilutions on microtitre plate wells. The plate was incubated at 37 °C for 2 h with gentle shaking. Wells were then emptied and washed twice with 300 µl of phosphate-buffered saline. Any remaining biofilm was stained with 1% crystal violet for 15 min, rinsed with water, allowed to dry and dissolved in 250 µl of 0.2% Triton X-100. Optical density at 595 nm was determined using a 96-well-plate spectrometer reader. Plots were produced using the Origin 2015 Sr software (OriginLab).

### Western blotting

The proteins were separated by electrophoresis in 18% SDS polyacrylamide gels and transferred onto an immuno-blot polyvinylidene difluoride membrane (Bio-Rad Laboratories) in Bio-Rad A buffer (25 mM Tris, pH 8.3, 192 mM glycine, with 20% methanol and 0.1% SDS) at 100 V or 350 mA for 1 h. The membrane was blocked with 5% skimmed milk in phosphate-buffered saline/Tween-20, incubated with primary anti-CsuA/B rabbit polyclonal antibodies (custom produced by Innovagen AB)^8^ followed by incubation with secondary IRDye 680RD-conjugated anti-rabbit goat antibodies (Li-Cor Biosciences). Protein bands were detected using the Odyssey system (Li-Cor Biosciences) and quantified using ImageJ (v.1.53k).

### Optical tweezers force measurements

To measure the biomechanical properties of Csu pili, we used a custom-made force-measuring optical tweezer set-up constructed around an inverted Olympus IX71 microscope (Olympus) equipped with a water-immersion objective (UPlanSApo60XWIR, ×60/1.2 NA; Olympus) and a 1,920 × 1,440 pixel CMOS camera (C11440-10C, Hamamatsu)^41^. To sample force data with a high signal-to-noise ratio with a minimal amount of drift, we used the Allan variance method to identify noise^42^. We used the power spectrum method to calibrate the trap by sampling the microspheres position at 131,072 Hz and averaging 32 consecutive datasets acquired for 0.25 s each. To extend a pilus, we moved the piezo stage at a constant speed of 50 nm s^−1^ and sampled the force and position at 50 Hz. To assess the mean contour length of the pilus quaternary structure, we buckled pili by reversing the piezo stage until the bead touched the bacterial cell wall. To estimate the persistence length and stretching modulus of pili, we fitted the initial force rise in region I with an extensible worm-like chain model^43^. From these values, we calculated the spring constant by dividing the stretch modulus with the contour length, and the flexural rigidity (bending stiffness) by multiplying the persistence length with the Boltzmann constant (4.1 pN nm). We calculated the plateau length by taking the difference between extension at the end of region I and the start of region III. Similarly, we calculated the plateau force by taking the mean of the force through the area between regions I and III. Finally, we estimated the length of region III using the method outlined previously^19^. All values are given as mean ± s.e.m.

### Temperature-depended folding transition analysis

Circular dichroism was measured using the Chirascan CD Spectrometer (Applied Photophysics) and a macro-cuvette 110-QS with 1 mm layer thickness (Hellma). The background for the spectra was first measured four times from the buffer (12.5 mM potassium phosphate at pH 7.0) before inserting the target protein at 0.150 mg ml^−1^ concentration. CD spectra at 20 °C were measured four times with the 195–260 nm wavelength range and using 1 nm intervals between each 3 s measurements. For the melting spectra, proteins were heated using 4 °C temperature ramping from 19 to 99 °C. Each spectrum was measured once after a 30 s temperature stabilization time using a wavelength range of 195–260 nm and 1 nm intervals between each 2 s measurement. The measurement of all melting spectra took 1 h 28 min. Each spectrum was smoothed by a factor of 4. Melting curves were recorded at a wavelength of 225 nm by heating the samples from 20 to 99 °C at the rate of 1 °C min^−1^. Circular dichroism was measured for 12 s at first every 1.0 °C and later every 0.5 °C with an error margin of 0.15 °C. Each recording took 1 h 19 min. The cuvette was purified of residual protein using 2 M potassium hydroxide between samples. The Curve Fitting function in the Chirascan user interface was used to fit melting data to the sigmoid curve + slope equation.

### Modelling of the assembly–secretion process

The Csu pilus models were constructed on the basis of the cryo-EM structure of the Csu pilus rod (this Article) and the crystal structures of CsuA/Bsc (PDB: 6FM5)^8^ and CsuC–CsuA/B chaperone–subunit complex (PDB: 5D6H)^24^. With no structure for the CsuD usher available, the models of the usher were based on the structures of the FimD usher from the classical CU pathway: the crystal structure (PDB: 3RFZ)^26^ and cryo-EM structures of conformers 1 and 2 (PDB codes 6E14 and 6E15, respectively^29^). The Phyre2 protein fold recognition server^44^ automatically modelled 92% of the full CsuD amino acid sequence on the basis of the structure of the entire FimD (conformer 1) with a confidence value of 100.0%. Models of the NTD of the usher at different steps of pilus secretion were produced based on the crystal structures of the NTD bound to preassembly complexes (PDB: 1ZE3 and 4B0M)^25,27^ and cryo-EM structures of the FimD conformers. Stereochemistry was analysed with *Coot*.

### Statistics and reproducibility

All data are presented as mean ± s.d. The cryo-EM image of the Csu pilus shown on Fig. 1a was selected from a dataset of around 100 micrographs and represents a typical image of a single pilus. The cryo-EM images of WT and Δ6 mutant surface-sheared pili material were selected from a dataset of about 20 micrographs recorded at different magnifications. The western blots on Fig. 4a and Extended Data Figs. 5b and 6a are representatives of three independent experiments. Melting of WT CsuA/Bsc was performed three times; CD spectra from one melting experiment are shown in Extended Data Fig. 6b. The biofilm quantification (Extended Data Fig. 7a) and CsuE exposure on the cell surface (Extended Data Fig. 7b) experiments were performed twice with smaller sets of mutants and controls examined several more times.

### Reporting summary

Further information on research design is available in the Nature Research Reporting Summary linked to this article.

## Online content

Any methods, additional references, Nature Research reporting summaries, source data, extended data, supplementary information, acknowledgements, peer review information; details of author contributions and competing interests; and statements of data and code availability are available at 10.1038/s41586-022-05095-0.

### Supplementary information

Supplementary Information Supplementary Figs. 1–3, Supplementary Tables 1 and 2, and legends for Supplementary Videos 1–3.

Reporting Summary

Supplementary Video 1 Structure of the Csu pilus rod. The contour level of the 3.4 Å resolution cryo-EM map shown on the video is 0.065. The model reveals the amino acid residues involved in the clinch interaction surface.

Supplementary Video 2 Model of opening-closing of the clinch contact. The linker between Gd and the globular domain of a pilin is flexible, but the A´-A´´ hairpin and Gd N-terminus restrict the rotation of pilins to an up-and-down movement. Therefore, the clinch contact confers the pilus rigidity and determines the trajectory of subunit movement upon clinch formation or pilus stretching.

Supplementary Video 3 Model of assembly-secretion mechanism in archaic systems. Each secretion step of Csu pilus translocates the pilus through the usher channel the length of exactly one subunit, and the exiting subunit forms a clinch with its preceding subunit. Note that the DSE process is not shown in the video. The DSE process occurs in a zip-in-zip-out fashion, in which the chaperone at the pilus base is replaced by the donor strand sequence of new-coming subunit in steps.

## Figures and Tables

**Extended Data Table 1:**
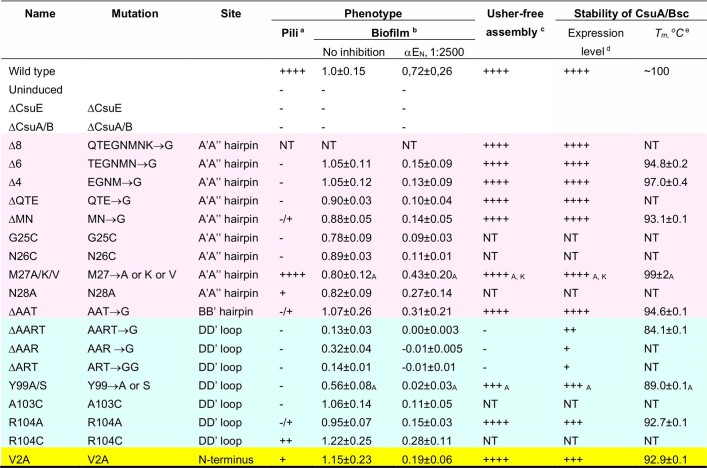
Summary of key properties of clinch-contact mutants

^a^Atomic force microscopy (AFM) (Extended Data Fig. 4a) and negative stain transmission electron microscopy (TEM) imaging of bacterial cells (Fig. 4c and Extended Data Fig. 4b). The number of plus signs indicates the amount of pili: ++++, amount similar to that of the wild type (WT); + to +++, a reduced number of WT-like pili; –/+, pilus-like structures observed on some bacterial cells but not others; –, no pili observed. NT, not tested. The total mass of extracted pilus material was evaluated by Western blotting using the anti-CsuA/Bsc antibody (see Extended Data Fig. 5b).

^b^Csu-mediated biofilms on plastics with or without anti-tip (αE_N_) antibody. The density of uninduced cells was subtracted from the biofilm cell density data (Fig. 4d and Extended Data Fig. 7a) and normalized to the biofilm density of WT cells in the absence of αE_N_. The result of biofilm inhibition by αE_N_ diluted 1:2500 times is shown (see Extended Data Fig. 7a for the full set of data).

^c^CsuC-assisted assembly of CsuA/B (Fig. 4b and Extended Data Fig. 6d). ++++, WT-like polymerization efficiency; +++, most subunits form DSC-polymers; -, not detected due to poor yield.

^d^Relative level of WT and mutant CsuA/Bsc in the *E. coli* periplasm (Fig. 4a and Extended Data Fig. 6a).

^e^Melting temperature (*T*_*m*_) of WT and mutant CsuA/Bsc obtained by measuring circular dichroism at different temperatures (Extended Data Fig. 6b,c). Data are mean ± s.d.

## Data Availability

The coordinates were deposited at the PDB under accession code 7ZL4. The corresponding cryo-EM map was deposited at the EMDB under accession code EMD-14777.
