# Supplementary material for: Archaic chaperone–usher pili self-secrete into superelastic zigzag springs
Source: Nature. 2022 Jul 19;609(7926):335–40. doi: 10.1038/s41586-022-05095-0 (PMC9452303; doi:10.1038/s41586-022-05095-0)
Supplement: Supplementary file 1 — Supplementary Figs. 1–3, Supplementary Tables 1 and 2, and legends for Supplementary Videos 1–3. [file 41586_2022_5095_MOESM1_ESM.pdf]

nature portfolio

https://doi.org/10.1038/s41586-022-05095-0

Supplementary information

Archaic chaperone–usher pili self-secrete into superelastic zigzag springs

In the format provided by the authors and unedited

SI Guide

Archaic chaperone-usher pili self-secrete into superelastic zigzag springs

Natalia Pakharukova, Henri Malmi, Minna Tuittila, Tobias Dahlberg, Debnath Ghosal, Yi-Wei Chang, Si Lhyam Myint[4], Sari Paavilainen, Stefan David Knight, Urpo Lamminmäki, Bernt Eric Uhlin, Magnus Andersson, Grant Jensen, Anton V. Zavialov

Supplementary information includes:

1. SI Figures 1-3 (pdf)
2. SI Tables 1 and 2 (pdf)
3. SI Videos 1-3 (separate files)

Legends for SI Figures

Supplementary Figure 1 | Original source images for the data obtained by electrophoretic separation. a and **b**, Original images of Western blots shown on Extended Data Fig. 5b (**a**) and Extended Data Fig. 6a (**b**). **c** and **d**, Original images of Coomassie Brilliant Blue R-250 stained gels shown on Fig. 4a (**c**) and Extended Data Fig. 6d (**d**). Rectangles indicate (approximately) how the gels were cropped for the final figures. Gels and blots were scanned with an Odyssey system (Li-Cor Biosciences) and selected scan regions may not contain the entire gel or blot. Western blots are also restricted by the size of the membrane used.

Supplementary Figure 2 | A representative cryo-EM micrograph of Csu pili. The micrograph was recorded on a 300 kV Titan Krios electron microscope (Thermo Scientific) at a magnification of 105,000 and a defocus of -3.0 μm.

Supplementary Figure 3 | Source images (enlarged versions) for Extended Fig. 4

Legends for SI Tables

Supplementary Table 1 | Cryo-EM data collection, refinement and validation statistics. The final pixel size is the corrected value obtained by calculating the range of cross-correlation coefficient values of the map with different voxel sizes to the refined model.

Supplementary Table 2 | Oligonucleotides and plasmids. List of oligonucleotides used to alter the plasmids to produce the various mutants studied in this article. The mutations are explained in Extended Data Table 1. Insertion and substitution sequences in the oligonucleotides are underlined.

Legends for SI Videos

Supplementary Video 1 | Structure of the Csu pilus rod. The contour level of the 3.4 Å resolution cryo-EM map shown on the video is 0.065. The model reveals the amino acid residues involved in the clinch interaction surface.

Supplementary Video 2 | Model of opening-closing of the clinch contact. The linker between Gd and the globular domain of a pilin is flexible, but the A´-A´´ hairpin and Gd N-terminus restrict the rotation of pilins to an up-and-down movement. Therefore, the clinch contact confers the pilus rigidity and determines the trajectory of subunit movement upon clinch formation or pilus stretching.

Supplementary Video 3 | Model of assembly-secretion mechanism in archaic systems. Each secretion step of Csu pilus translocates the pilus through the usher channel the length of exactly one subunit, and the exiting subunit forms a clinch with its preceding subunit. Note that the DSE process is not shown in the video. The DSE process occurs in a zip-in-zip-out fashion, in which the chaperone at the pilus base is replaced by the donor strand sequence of new-coming subunit in steps.

Supplementary information

Archaic chaperone-usher pili self-secrete into superelastic zigzag springs

Natalia Pakharukova, Henri Malmi, Minna Tuittila, Tobias Dahlberg, Debnath Ghosal, Yi-Wei Chang, Si Lhyam Myint, Sari Paavilainen, Stefan David Knight, Urpo Lamminmäki, Bernt Eric Uhlin, Magnus Andersson, Grant Jensen, Anton V. Zavialov

Supplementary figures 1-3

Supplementary Figure 1

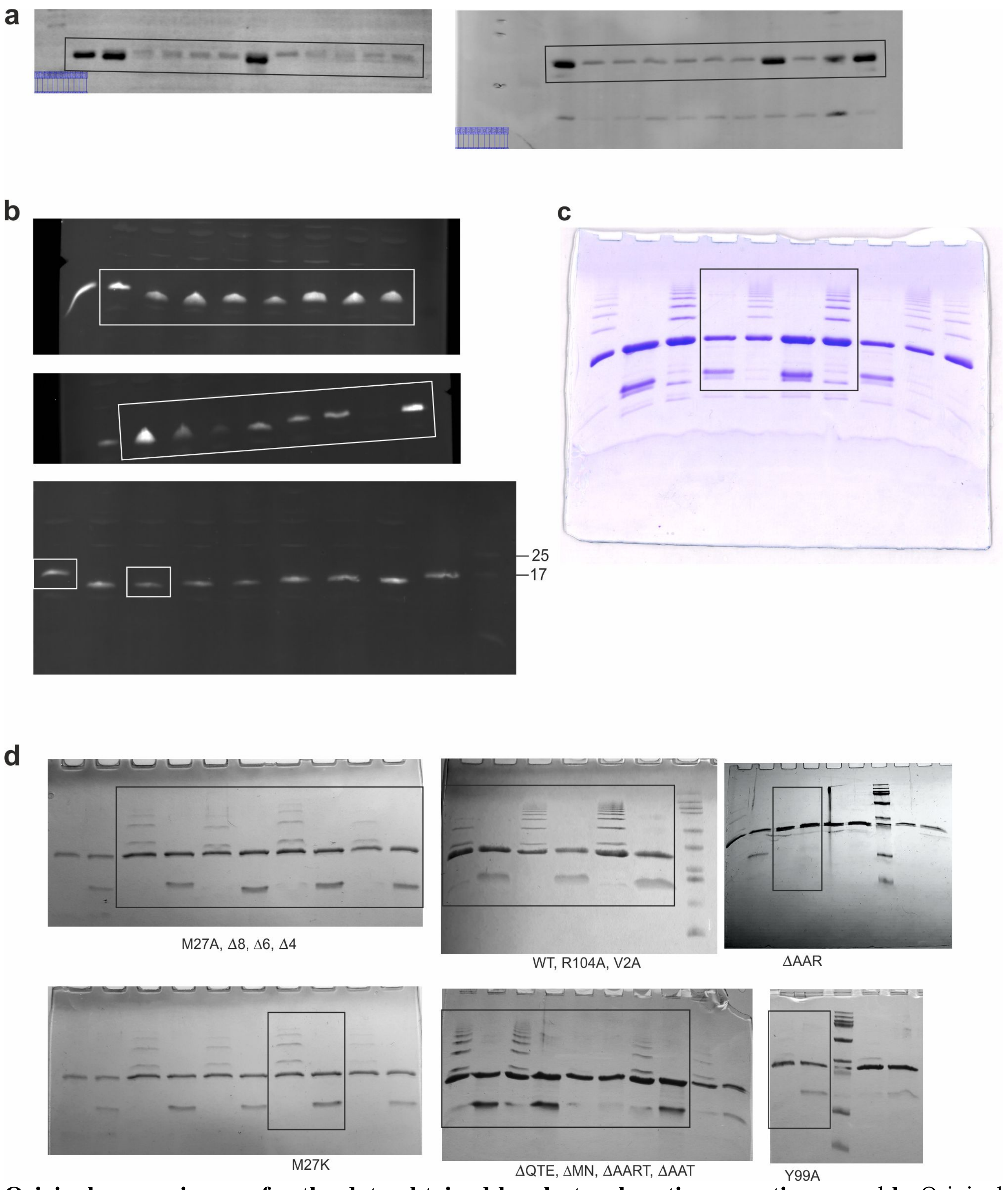

Original source images for the data obtained by electrophoretic separation. a and **b**, Original images of Western blots shown on Extended Data Fig. 5b (**a**) and Extended Data Fig. 6a (**b**). **c** and **d**, Original images of Coomassie Brilliant Blue R-250 stained gels shown on Fig. 4a (**c**) and Extended Data Fig. 6d (**d**). Rectangles indicate (approximately) how the gels were cropped for the final figures. Gels and blots were scanned with an Odyssey system (Li-Cor Biosciences) and selected scan regions may not contain the entire gel or blot. Western blots are also restricted by the size of the membrane used.

Supplementary Figure 2

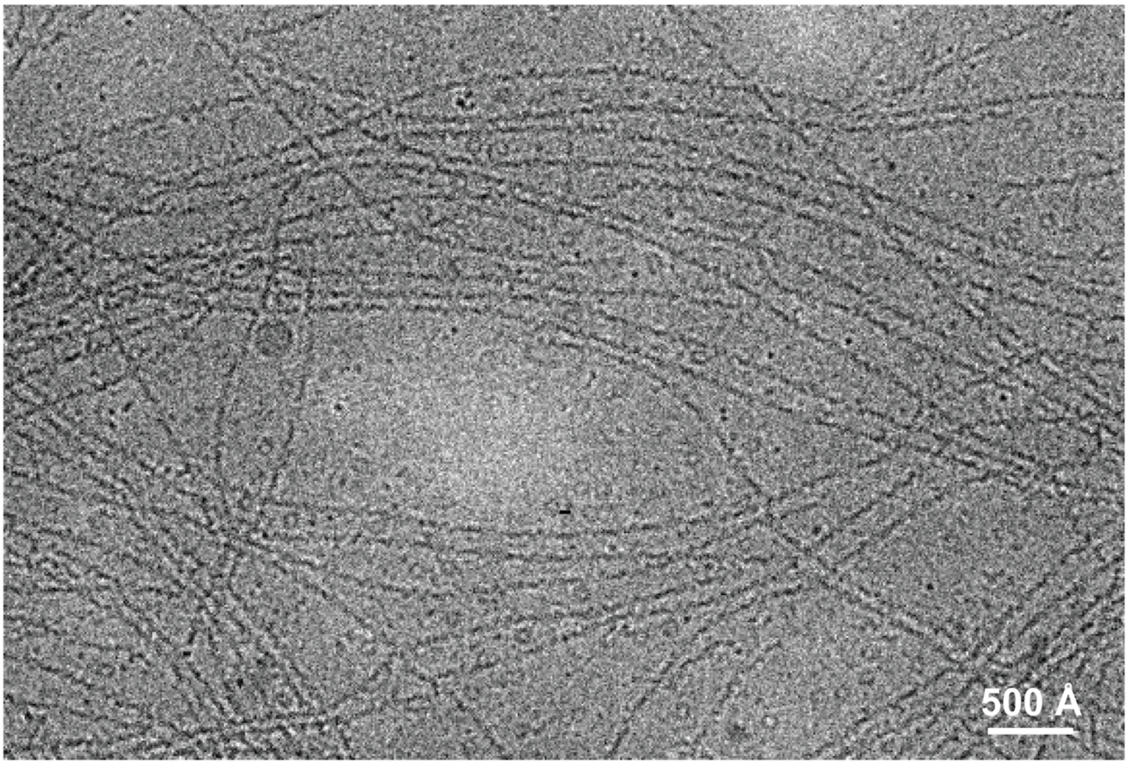

A representative cryo-EM micrograph of Csu pili. The micrograph was recorded on a 300 kV Titan Krios electron microscope (Thermo Scientific) at a magnification of 105,000 and a defocus of -3.0 μm.

Supplementary Figure 3

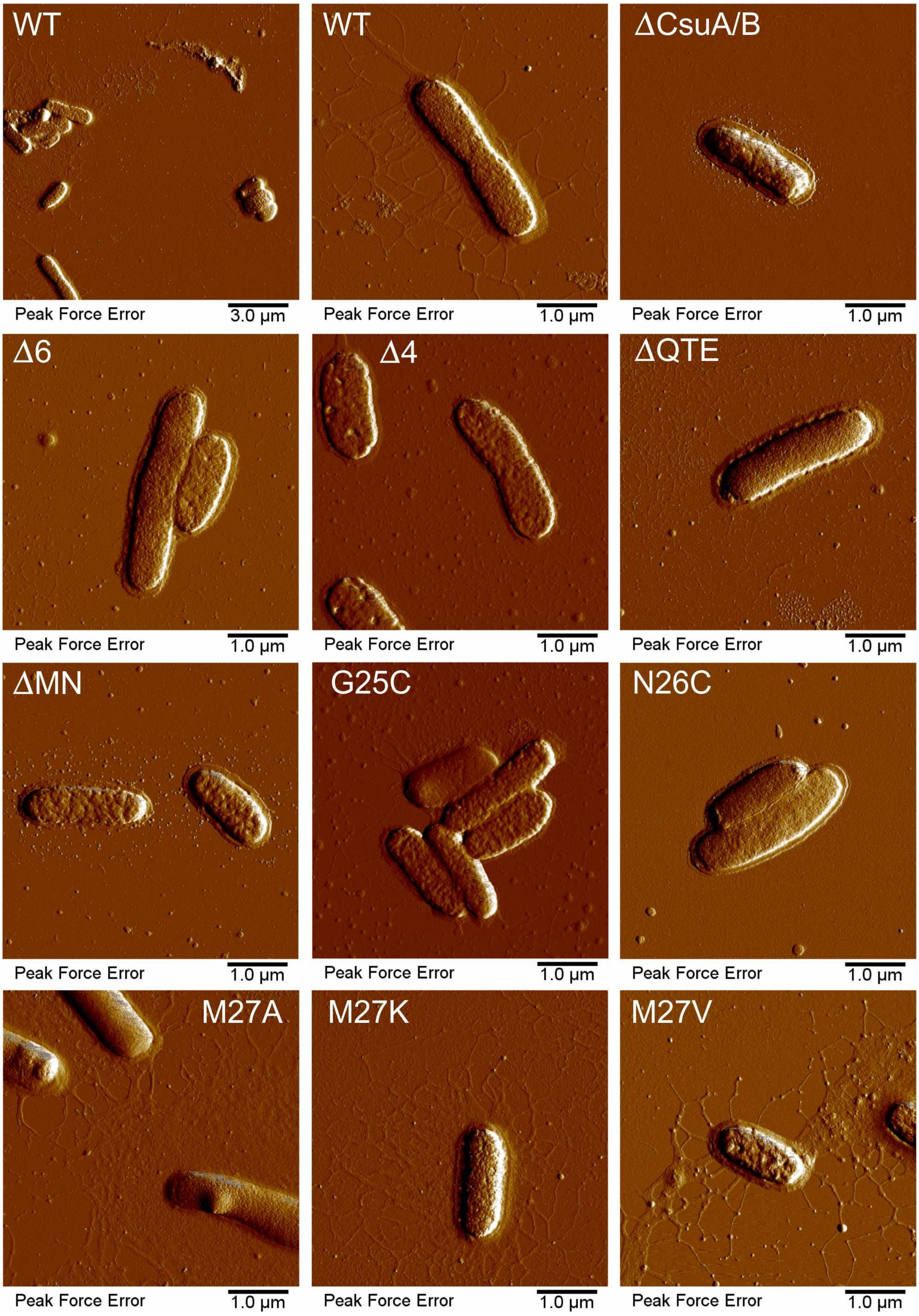

Continues on the next page

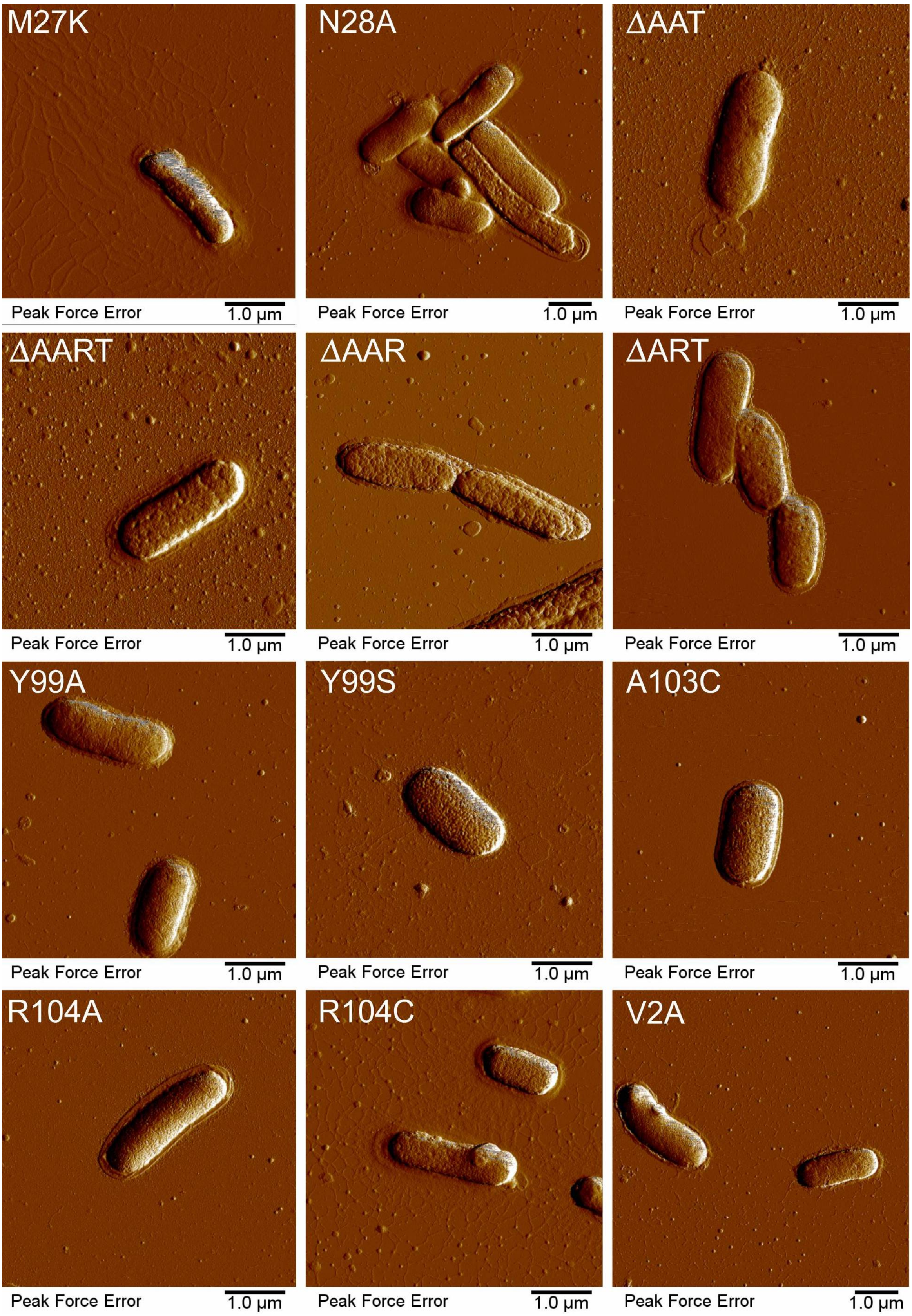

Continues on the next page

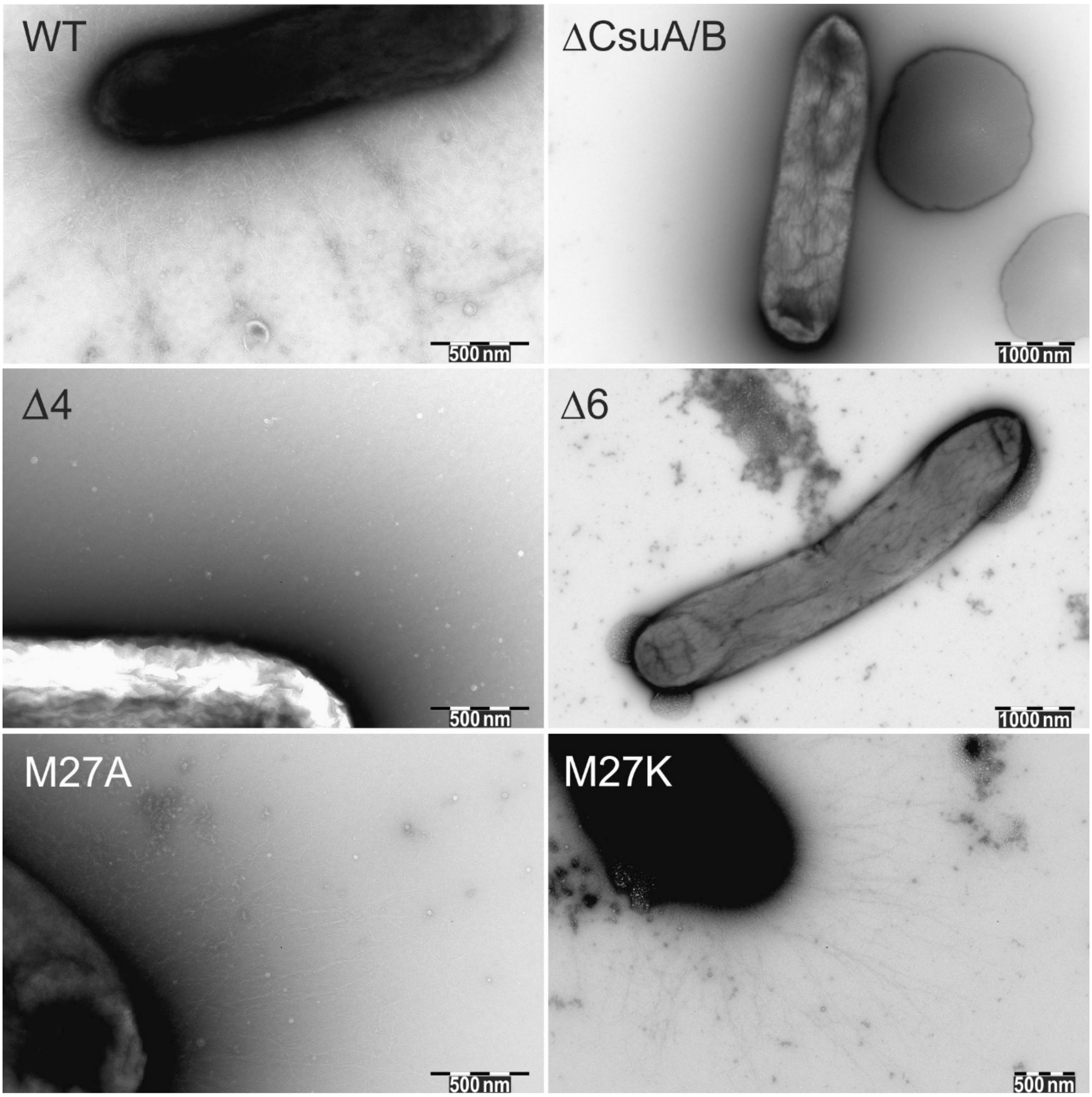

Source images (enlarged versions) for Extended Fig. 4.

Supplementary Tables 1-2

Supplementary Table 1 | Cryo-EM data collection, refinement and validation statistics

	Csu pilus rod segment (EMDB-14777) (PDB 7ZL4)
**Data collection and processing**	
Magnification	105,000x
Voltage (kV)	300
Electron exposure (e–/Å^2)	60
Defocus range (μm)	-1.0 to -3.0
Pixel size (Å)	0.433 (collection)<br>1.2949 (final)
Symmetry imposed	Helical
Helical twist	-152.8°
Helical rise	27.97 Å
Initial segment images (no.)	480,064
Final segment images (no.)	255,833
Map resolution (Å)	3.42
FSC threshold	0.143
Map resolution range (Å)	3.42-4.2
**Refinement**	
Initial model used (PDB code)	6FM5
Model resolution (Å)	3.62
FSC threshold	0.5
Map sharpening *B* factor (Å^2)	-144.18
Model composition	
Non-hydrogen atoms	3387
Protein residues	465
Ligands	---
B factors (Å^2)	
Protein (min/max/mean)	49.50/182.85/110.98
Ligand	---
R.m.s. deviations	
Bond lengths (Å)	0.007
Bond angles (°)	1.388
Validation	
MolProbity score	1.11
Clashscore	3.15
Poor rotamers (%)	0.00
Ramachandran plot	
Favored (%)	98.25
Allowed (%)	1.75
Disallowed (%)	0.00

Supplementary Table 2 | Oligonucleotides and plasmids

Mutation [a]	Forward and reverse oligonucleotides (5'→3') [b]	Generated plasmids
ΔCsuE	TCATGGCAAAGATACCTCGTGA<br>TAAAAGCTGTTTTATATAGGAGATAAAAAG	pBAD-Csu(A/B)ABCD(ΔE)
ΔCsuA/B	GAATTCCGTTAATTCCTCCTGTTAG<br>GAGTAGCAGGTTTGCTCAAATATG	pBAD-Csu(ΔA/B)ABCDE
Δ8	CACTACCACCTACAGTACAGCC<br>GATTTGGTACTTTAAATTTTGGTAAAACTTC	pET101-6HCsuA/Bdsc-Δ8,<br>pET101-CsuC6H-CsuA/B-Δ8
Δ6	CTTGACTACCACCTACAGTACAG<br>GTAAGTTTGGTACTTTAAATTTTGGTAAAAC	pBAD-Csu(A/B-Δ6)ABCDE, pET101-6HCsuA/Bdsc-Δ6,<br>pET101-CsuC6H-CsuA/B-Δ6
Δ4	CAGTTTGACTACCACCTACAGTAC<br>GTAACAAGTTTGGTACTTTAAATTTTGGTAAAAC	pBAD-Csu(A/B-Δ4)ABCDE, pET101-6HCsuA/Bdsc-Δ4,<br>pET101-CsuC6H-CsuA/B-Δ4
ΔQTE	CACTACCACCTACAGTACAGCC<br>GTGGAAATATGAACAAGTTTGGTACTTTAAATTTTG	pBAD-Csu(A/B-ΔQTE)ABCDE, pET101-6HCsuA/Bdsc-ΔQTE,<br>pET101-CsuC6H-CsuA/B-ΔQTE
ΔMN	CATTTCCTTCAGTTTGACTACCAC<br>GTAAGTTTGGTACTTTAAATTTTGGTAAAAC	pBAD-Csu(A/B-ΔMN)ABCDE, pET101-6HCsuA/Bdsc-ΔMN,<br>pET101-CsuC6H-CsuA/B-ΔMN
G25C	CATTCAGTTTGACTACCACCTACAGTAC<br>TAATATGAACAAGTTTGGTACTTTAAATTTTGG	pBAD-Csu(A/B-G25C)ABCDE
N26C	TGACTACCACCTACAGTAC<br>AACTGAAGGATGTATGAACAAGTTTGGTACTTTAAATTTG	pBAD-Csu(A/B-N26C)ABCDE
M27A	GCATTTCCTTCAGTTTGACTACCACCTAC<br>GAACAAGTTTGGTACTTTAAATTTTGGTAAAAC	pBAD-Csu(A/B-M27A)ABCDE, pET101-6HCsuA/Bdsc-M27A,<br>pET101-6HCsuA/Bdsc-M27A
M27K	TTATTTCCTTCAGTTTGACTACCACCTAC<br>GAACAAGTTTGGTACTTTAAATTTTGGTAAAAC	pBAD-Csu(A/B-M27K)ABCDE, pET101-6HCsuA/Bdsc-M27K,<br>pET101-CsuC6H-CsuA/B-M27K
M27V	GTTTGACTACCACCTACAG<br>TGAAGGAAATGTGAACAAGTTTGGTAC	pBAD-Csu(A/B-M27V)ABCDE
M27C	GTTTGACTACCACCTACAGTACAG<br>TGAAGGAAATTGTAACAAGTTTGGTAC	pBAD-Csu(A/B-M27C)ABCDE, pET101-6HCsuA/Bdsc-M27C,<br>pET101-CsuC6H-CsuA/B-M27C
N28A	TCAGTTTGACTACCACCTACAGTACAG<br>AGGAAATATGGCCAAGTTTGGTACTTTAAATTTTG	pBAD-Csu(A/B-N28A)ABCDE
ΔAAT	CTGAAGCAACTTCAGCTGTTAATAC<br>GTGGTGGCAATATTTCTGTGACTTG	pBAD-Csu(A/B-ΔAAT)ABCDE, pET101-6HCsuA/Bdsc-ΔAAT,<br>pET101-CsuC6H-CsuA/B-ΔAAT
ΔAART	CATCACGATAAACGTTATATGCAACTAC<br>GTAACCTTTATGTTGTAAACCAACCAC	pBAD-Csu(A/B-ΔAART)ABCDE, pET101-6HCsuA/Bdsc-ΔAART,<br>pET101-CsuC6H-CsuA/B-ΔAART
ΔAAR	CATCACGATAAACGTTATATGCAACTAC<br>GTACAAACCTTTATGTTGTAAACCAACCAC	pBAD-Csu(A/B-ΔAAR)ABCDE, pET101-6HCsuA/Bdsc-ΔAAR,<br>pET101-CsuC6H-CsuA/B-ΔAAR
ΔART	CTCCAGCATCACGATAAACGTTATATG<br>GTAACCTTTATGTTGTAAACCAACCAC	pBAD-Csu(A/B-ΔART)ABCDE, pET101-6HCsuA/Bdsc-ΔART,<br>pET101-CsuC6H-CsuA/B-ΔART
Y99A	CAACGTTATATGCAACTACATCAGCAG<br>CTCGTGATGCTGCACGTACAAAC	pBAD-Csu(A/B-Y99A)ABCDE, pET101-6HCsuA/Bdsc-Y99A,<br>pET101-CsuC6H-CsuA/B-Y99A
Y99S	This substitution was generated by accident<br>with oligos designed to produce Y99A	pBAD-Csu(A/B-Y99S)ABCDE
A103C	TAAACGTTATATGCAACTACATCAGCAGAAGCAG<br>TCGTGATGCTTGTCGTACAAACCTTTATGTTG	pBAD-Csu-ΔCsuA/B-A103C
R104A	CTGCAGCATCACGATAAACGTTATATG<br>CTACAAACCTTTATGTTGTAAACCAACCAC	pBAD-Csu(A/B-R104A)ABCDE, pET101-6HCsuA/Bdsc-R104A,<br>pET101-CsuC6H-CsuA/B-R104A
R104C	CGATAAACGTTATATGCAACTAC<br>TGATGCTGCATGTACAAACCT	pBAD-Csu(A/B-R104C)ABCDE
V2A	AGTATTTACCGCATAACCAGCAAC<br>CAAGCAGCTGCTACTGGTCAG	pBAD-Csu(A/B-V2A)ABCDE, pET101-CsuC6H-CsuA/B-V2A
V2A for<br>CsuA/Bsc	CATTTTGTTTGTTATCGAAATTTACAGTGACTAATAG<br>GAGGTGCTGCTACTGGTCAGG	pET101-6HCsuA/Bdsc-V2A

[a] Mutations are explained in Extended Data Table 1. [b] Insertions and substitutions are underlined
