# Supplementary material for: Archaic chaperone–usher pili self-secrete into superelastic zigzag springs
Source: Nature. 2022 Jul 19;609(7926):335–40. doi: 10.1038/s41586-022-05095-0 (PMC9452303; doi:10.1038/s41586-022-05095-0)
Supplement: Supplementary file 2 — Reporting Summary [file 41586_2022_5095_MOESM2_ESM.pdf]

nature portfolio

Corresponding author(s): Anton V. Zavialov

Last updated by author(s): Jun 28, 2022

Reporting Summary

Nature Portfolio wishes to improve the reproducibility of the work that we publish. This form provides structure for consistency and transparency in reporting. For further information on Nature Portfolio policies, see our Editorial Policies and the Editorial Policy Checklist.

Statistics

For all statistical analyses, confirm that the following items are present in the figure legend, table legend, main text, or Methods section.

n/a	Confirmed	
☐	☒	The exact sample size (*n*) for each experimental group/condition, given as a discrete number and unit of measurement
☒	☐	A statement on whether measurements were taken from distinct samples or whether the same sample was measured repeatedly
☒	☐	The statistical test(s) used AND whether they are one- or two-sided<br>*Only common tests should be described solely by name; describe more complex techniques in the Methods section.*
☒	☐	A description of all covariates tested
☒	☐	A description of any assumptions or corrections, such as tests of normality and adjustment for multiple comparisons
☐	☒	A full description of the statistical parameters including central tendency (e.g. means) or other basic estimates (e.g. regression coefficient) AND variation (e.g. standard deviation) or associated estimates of uncertainty (e.g. confidence intervals)
☒	☐	For null hypothesis testing, the test statistic (e.g. *F*, *t*, *r*) with confidence intervals, effect sizes, degrees of freedom and *P* value noted<br>*Give P values as exact values whenever suitable.*
☒	☐	For Bayesian analysis, information on the choice of priors and Markov chain Monte Carlo settings
☒	☐	For hierarchical and complex designs, identification of the appropriate level for tests and full reporting of outcomes
☒	☐	Estimates of effect sizes (e.g. Cohen's *d*, Pearson's *r*), indicating how they were calculated

Our web collection on statistics for biologists contains articles on many of the points above.

Software and code

Policy information about availability of computer code

Data collection	SerialEM v3.6 software was used for automated cryo-EM data collection
Data analysis	Cryo-EM data processing and 3D reconstruction was performed using MotionCor2 v1.2.3, RELION 3.0.8, CTFFind v4.1.13, SPRING v0.86, and EMAN 2. PHENIX v1.8.2 and MolProbity v4.5.1 were used for model refinement and validation. Coot v0.9.4 and UCSF Chimera v1.15 were used for model building. Model prediction was ran by PHYRE2. Biochemical and biophysical data were analyzed using ImageJ v1.53k, NanoScope software v1.8, Chirascan CD Spectrometer analysis software (Applied Photophysics), and Origin 2015 Sr (OriginLab Corporation).

For manuscripts utilizing custom algorithms or software that are central to the research but not yet described in published literature, software must be made available to editors and reviewers. We strongly encourage code deposition in a community repository (e.g. GitHub). See the Nature Portfolio guidelines for submitting code & software for further information.

Data

Policy information about availability of data

All manuscripts must include a data availability statement. This statement should provide the following information, where applicable:

- Accession codes, unique identifiers, or web links for publicly available datasets
- A description of any restrictions on data availability
- For clinical datasets or third party data, please ensure that the statement adheres to our policy

The coordinates were deposited to the PDB with the accession code 7ZL4. The cryo-EM map was deposited in the EMDB with accession code EMD-14777. PDB codes for the various structures reported in this manuscript are 6FM5, 5D6H, 3RFZ, 6E14, 6E15, 1ZE3, and 4B0M. All constructs used in this study can be obtained on request to AVZ

Field-specific reporting

Please select the one below that is the best fit for your research. If you are not sure, read the appropriate sections before making your selection.

☒ Life sciences ☐ Behavioural & social sciences ☐ Ecological, evolutionary & environmental sciences

For a reference copy of the document with all sections, see nature.com/documents/nr-reporting-summary-flat.pdf

Life sciences study design

All studies must disclose on these points even when the disclosure is negative.

Sample size	Sample sizes were not predetermined based on statistical methods, but were chosen according to the standards of the field (at least three independent biological replicates). For the cryoEM analysis, the filaments were manually picked from 602 selected micrographs. After autopicking of helical filaments, a total of 480,064 segments were extracted.
Data exclusions	No data was systematically excluded from analysis.
Replication	A minimum of three independent experiments were performed. All attempts at replication succeeded and no data were excluded from analysis. For cryo-EM, data were collected several times using multiple samples prepared in different time.
Randomization	Not relevant because no human or animal subjects were used in the study. Randomization is not generally used in this field.
Blinding	Experimental results were quantitative and did not require subjective analysis. Therefore, blinding was not performed.

Reporting for specific materials, systems and methods

We require information from authors about some types of materials, experimental systems and methods used in many studies. Here, indicate whether each material, system or method listed is relevant to your study. If you are not sure if a list item applies to your research, read the appropriate section before selecting a response.

Materials & experimental systems

n/a	Involved in the study	
☐	☒	Antibodies
☒	☐	Eukaryotic cell lines
☒	☐	Palaeontology and archaeology
☒	☐	Animals and other organisms
☒	☐	Human research participants
☒	☐	Clinical data
☒	☐	Dual use research of concern

Methods

n/a	Involved in the study	
☒	☐	ChIP-seq
☒	☐	Flow cytometry
☒	☐	MRI-based neuroimaging

Antibodies

Antibodies used	Anti-CsuA/B and anti-CsuEN rabbit polyclonal antibodies were custom produced by Innovagen AB for our previous studies; RDye 680RD-conjugated anti-rabbit goat antibody RRID AB_10956166 (Li-Cor Biosciences)
Validation	Anti-CsuA/B and anti-CsuEN rabbit polyclonal antibodies were validated with ELISA and Western Blotting in our lab and with ELISA by Innovagen AB using purified CsuA/Bsc and CsuEN proteins in previous studies. RDye 680RD-conjugated anti-rabbit goat antibody was tested by dot blot and and/or solid-phase adsorbed for minimal cross-reactivity with human, mouse, rat, sheep, and chicken serum proteins, but may cross-react with immunoglobulins from other species. The conjugate has been specifically tested and qualified for Western blot and In-Cell Western™ assay applications (https://www.licor.com/bio/reagents/irdye-680rd-goat-anti-rabbit-igg-secondary-antibody)
